# Solvent‐Free Synthesis of Ultrabright Carbon Dots With Near‐Unity Quantum Yield for High‐Performance Light‐Emitting Diodes

**DOI:** 10.1002/advs.77885

**Published:** 2026-09-27

**Authors:** Hyeonjin Park, Su Hwan Lee, Sejeong Seo, Soye Park, Soo min Ji, Hyunda Jo, Sehyeon Park, Young‐Hoon Kim, Woosung Kwon

**Affiliations:** ^1^ Department of Chemical and Biological Engineering Sookmyung Women's University Seoul Republic of Korea; ^2^ Department of Energy Engineering Hanyang University Seoul Republic of Korea; ^3^ Department of Battery Engineering Hanyang University Seoul Republic of Korea; ^4^ Institute of Advanced Materials and Systems Sookmyung Women's University Seoul Republic of Korea; ^5^ Trion Co. Ltd. Uiwang‐si Gyeonggi‐do Republic of Korea

**Keywords:** carbon dots, electroluminescence, intra‐particle energy funneling, solvent‐free carbonization, surface passivation

## Abstract

Conventional liquid‐phase syntheses of carbon dots (CDs) often involve complex solvent‐precursor interactions that induce structural and spectral heterogeneities, severely limiting their optoelectronic performance. Herein, we report a highly controlled, solvent‐free, single‐precursor strategy utilizing 2,6‐diaminonaphthalene to synthesize ultrabright, highly uniform green‐emissive CDs (DAN‐CDs). Through comprehensive thermal analyses and kinetic modeling, we elucidate a spatiotemporally resolved, dual‐pathway formation mechanism: a nitrogen‐doped core initially assembles within an oxygen‐shielding autogenic precursor melt, followed by controlled, oxygen‐mediated surface passivation as the melt barrier attenuates. This self‐limiting oxidative capping thoroughly suppresses non‐radiative structural defects. Photophysical analyses uncover an efficient intra‐particle energy funneling process from the light‐harvesting core to these surface traps, yielding excitation‐independent green emission with a narrow bandwidth (< 60 nm) and a near‐unity photoluminescence quantum yield of 91%. When integrated into self‐emissive light‐emitting diodes (LEDs), the devices deliver pure green electroluminescence. Notably, the peak external quantum efficiency (EQE) of 2.2% achieved herein through the solvent‐free approach stands as the highest value reported to date among LEDs based on green‐emissive CDs synthesized via thermal carbonization. These results conclusively underscore the strong potential of rationally designed DAN‐CDs as highly efficient, environmentally benign, and heavy‐metal‐free emitters for next‐generation optoelectronics and display applications.

## Graphical abstract

A rational, solvent‐free synthesis utilizing 2,6‐diaminonaphthalene produces ultrabright green‐emissive carbon dots. Regulated by a self‐limiting oxidative capping mechanism, excitons undergo ultrafast intra‐particle funneling from the nitrogen‐doped carbon core to emissive surface traps. This highly efficient architecture yields a near‐unity photoluminescence quantum yield (91%) and a record‐breaking 2.2% external quantum efficiency in light‐emitting diodes.

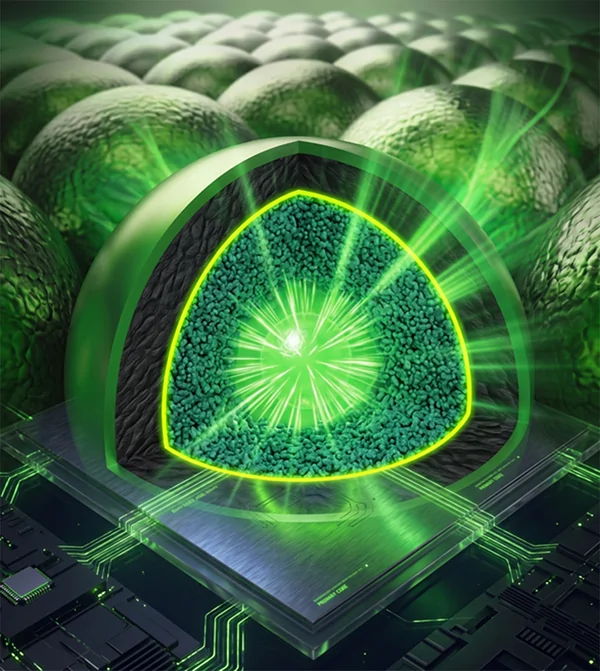

## 1 Introduction

Carbon dots (CDs) have attracted immense attention as a versatile class of luminescent nanomaterials owing to their exceptional photostability, low toxicity, and inherent biocompatibility [1, 2, 3, 4]. Driven by these compelling attributes, CDs have been extensively investigated across a broad spectrum of applications, ranging from bioimaging and chemical sensing to photocatalysis and optoelectronics [5, 6, 7, 8]. Within the optoelectronic domain, CDs featuring high photoluminescence (PL) efficiencies and narrow emission bandwidths are particularly sought after for next‐generation light‐emitting diodes (LEDs). Recent rapid advancements in this field have demonstrated that intense brightness and stringent color purity are prerequisites for practical commercial viability, prompting a surge of innovations in precursor engineering, interface modification, and device architectures aimed at maximizing electroluminescence (EL) [9, 10, 11, 12, 13, 14].

The physicochemical and optical properties of bottom‐up synthesized CDs are fundamentally governed by both the choice of molecular precursors and the specific reaction conditions [15, 16]. Among the various synthetic approaches, solvent‐based methods, such as hydrothermal and solvothermal reactions, are predominantly utilized due to their procedural simplicity, broad precursor compatibility, and facile scalability [17, 18]. However, these liquid‐phase methods inherently involve complex solvent‐precursor interactions and multiple concurrent reaction pathways, which often exacerbate the structural and chemical heterogeneity of the resulting CDs [19, 20]. Consequently, the formation of diverse, ill‐defined defect or surface states renders the electronic structure of CDs highly unpredictable. This structural ambiguity often introduces detrimental non‐radiative trap centers that severely quench luminescence [21, 22]. Furthermore, when combined with broad size distributions, it typically leads to widened emission spectra, undesirable excitation‐dependent luminescence, and formidable challenges in engineering specific excited‐state dynamics [23]. Overcoming such structural and spectral heterogeneities remains a critical bottleneck in the development of CDs with the highly pure, well‐defined optical outputs required for high‐performance optoelectronics.

To circumvent these limitations, simplifying the synthetic environment has emerged as an effective strategy to exert precise chemical control over the formation of emissive domains. In this context, solvent‐free carbonization utilizing a single molecular precursor provides a confined and regulated reaction system by eliminating unpredictable solvent effects and minimizing the variables governing CD formation. This streamlined reaction environment facilitates rigorous regulation of the carbonization process, effectively narrowing the distribution of chemical motifs and localized emissive centers in the final products [24, 25]. Consequently, the reduction in structural complexity simultaneously mitigates non‐radiative exciton quenching and narrows the emissive pathways, yielding CDs defined by both superior radiative efficiency and color purity.

Among the available molecular precursors, aromatic diamines [26, 27] represent attractive candidates. Their extended *π*‐conjugated frameworks and reactive nitrogen‐rich functional groups promote the direct formation of heteroatom‐doped carbon architectures during thermal treatment. While solid‐state carbonization systems utilizing single‐ring aromatic diamines (such as o‐ or p‐phenylenediamine) have been successfully established [24, 28], the restricted dimensions and geometric constraints of these simple benzene derivatives can inherently limit the extent of ordered polyaromatic condensation, restricting their ultimate optoelectronic potential (Table S1). To fundamentally overcome this structural limitation and unlock the highly pure emissive states required for advanced optoelectronics, we introduce a rational topological design strategy utilizing 2,6‐diaminonaphthalene (DAN) [29]. Compared to single‐ring diamines, DAN provides an extended, rigid polycyclic aromatic backbone coupled with highly reactive primary amine groups positioned in a symmetric, linear configuration. This specific molecular topology minimizes steric hindrance during thermal treatment, facilitating a highly ordered and extended polyaromatic step‐growth condensation. These structural pre‐organization characteristics make DAN a highly effective building block for constructing robust, nitrogen‐doped carbon core frameworks with minimal non‐radiative defect sites. Furthermore, the progressive thermal condensation generates reactive intermediate edge sites available for further modification. Notably, when carbonization is conducted in air, ambient oxygen readily interacts with these newly formed peripheral sites, enabling tailored, oxygen‐mediated surface functionalization. Optically, the inherent nitrogen atoms construct a nitrogen‐doped conjugated core that defines the intrinsic optical bandgap, while the highly electronegative oxygen‐mediated surface functionalities act as efficient, lower‐energy emissive traps [30, 31].

Herein, we report the rational design and synthesis of ultrabright CDs (denoted as DAN‐CDs) via the solvent‐free, single‐precursor carbonization of DAN. By confining the synthesis to this streamlined paradigm, we effectively suppress the heterogeneous chemical motifs and non‐radiative trap states that typically limit CD performance. Indeed, the synthesized DAN‐CDs exhibit robust, excitation‐independent green emission, characterized by an absolute PL quantum yield (PLQY) of > 90% and a narrow full‐width at half‐maximum (FWHM) of < 60 nm. To uncover the mechanistic origins of this highly efficient emission, we systematically compare samples synthesized under ambient air (oxidative) and inert nitrogen atmospheres, investigating the critical role of oxygen in driving intermediate surface functionalization and forming well‐defined lower‐energy states. Furthermore, we evaluate the temperature‐dependent structural evolution of the CDs to determine the optimal balance between aromatic condensation and surface passivation. Finally, to demonstrate the practical viability of this approach, the optimized DAN‐CDs are integrated into proof‐of‐concept LED devices, highlighting their potential for advanced optoelectronic and display technologies.

## 2 Materials and Methods

### 2.1 Materials

2,6‐Diaminonaphthalene (DAN, 98%) was purchased from Tokyo Chemical Industry Co., Ltd. Toluene (TOL, anhydrous, 99.8%), tetrahydrofuran (THF, anhydrous, 99.9%), acetonitrile (ACN, anhydrous, 99.8%), N,N‐dimethylformamide (DMF, anhydrous, 99.8%), dimethyl sulfoxide (DMSO, anhydrous, 99.9%), 1‐pentanol (PeOH, 99%), 1‐butanol (BuOH, 99%), 1‐propanol (PrOH, anhydrous, 99.9%), methanol (MeOH, 99.9%), and ethanol (EtOH, anhydrous, 99.5%) were obtained from Sigma–Aldrich. Deuterium dimethyl sulfoxide (DSMO‐d_6_) was purchased from Cambridge Isotope Laboratories, Inc. All chemicals were used as received without further purification. Cellulose ester (CE) dialysis tubes (molecular weight cut‐off: 100–500 Da) were purchased from Spectra/Por Biotech. Polytetrafluoroethylene (PTFE) membrane filters (pore size: 0.2 µm) were obtained from Whatman. Deionized water (DW) was obtained from a Milli‐Q purification system (Millipore). Poly(3,4‐ethylenedioxythiophene)/poly(styrenesulfonate) (PEDOT:PSS) was purchased from Heraeus. 4,4',4‐Tris(carbazol‐9‐yl)triphenylamine (TCTA) and 2,2′,2″‐(1,3,5‐benzinetriyl)‐tris(1‐phenyl‐1‐H‐benzimidazole) (TPBI) were purchased from Organic Semiconductor Materials (OSM).

### 2.2 Synthesis of DAN‐CDs via Solvent‐Free Carbonization

The DAN‐CDs were synthesized via the solvent‐free carbonization of DAN. Briefly, 800 mg of the DAN precursor was placed in an alumina crucible and heated in a muffle furnace under an ambient air atmosphere. The sample was heated to 300°C at a ramping rate of 5°C/min and maintained at this temperature for ∼24 h. After naturally cooling to room temperature, the obtained solid product was dissolved in TOL and dialyzed against fresh TOL for at least one week using a CE 100–500 Da dialysis tube. The solvent was subsequently removed by rotary evaporation, and the remaining solid sample was stored in a desiccator until measurements were performed. The final product yield was approximately 20%.

To investigate the structural evolution and the effect of temperature on the optical properties, additional samples were prepared at varying carbonization temperatures (100°C, 200°C, and 400°C) while keeping all other parameters constant. Furthermore, to elucidate the specific role of oxygen during synthesis, a control sample (denoted as DAN‐CDs‐N_2_) was prepared by heating DAN to 300°C at a ramping rate of 5°C/min under an inert nitrogen atmosphere for 24 h. The resulting product was purified using an identical procedure and compared with the DAN‐CDs synthesized under oxidative (air) conditions.

### 2.3 Material Characterization

Thermogravimetric analysis (TGA) and differential scanning calorimetry (DSC) were performed using a TA Instruments SDT650 analyzer. DAN was heated from room temperature to 600°C at a heating rate of 10°C/min under oxygen and nitrogen atmospheres, respectively. For transmission electron microscopy (TEM) analysis, the DAN‐CDs were dispersed in TOL (5 mg/mL), and the dispersion was drop‐cast onto a carbon‐supported 200‐mesh copper grid. TEM observations were conducted using a JEOL JEM‐F200 microscope, and high‐resolution TEM (HR‐TEM) imaging was performed on a JEOL JEM‐200F NEOARM microscope equipped with a spherical aberration (Cs) corrector. For dynamic light scattering (DLS) analysis, the DAN‐CDs were dispersed in TOL (1 mg/mL), and their hydrodynamic size distribution was determined using a Horiba SZ‐100 particle size analyzer. X‐ray diffraction (XRD) patterns were acquired using a Bruker D8 Advance diffractometer. Raman spectroscopy was performed on an Alvatek XperRam S spectrometer with a 532 nm excitation laser. Fourier transform infrared (FTIR) spectroscopy was performed using a Thermo Scientific Nicolet iS50 FTIR spectrometer equipped with an attenuated total reflection (ATR) accessory. The ^1^H nuclear magnetic resonance (NMR) spectra of the DAN precursor, DAN‐CDs, and DAN‐CDs‐N_2_ dissolved in DMSO‐d_6_ were acquired using a Bruker Avance III HD500 spectrometer (500 MHz). X‐ray photoelectron spectroscopy (XPS) measurements were conducted on a Thermo Scientific K‐Alpha system equipped with a monochromatic Al Kα X‐ray source (1486.6 eV).

### 2.4 Optical Characterization

For steady‐state spectroscopic measurements, the DAN precursor, DAN‐CDs, and DAN‐CDs‐N_2_ were dispersed in TOL (0.05 mg/mL) and analyzed in Hellma Analytics 10 mm × 10 mm QS‐grade quartz cuvettes (111‐QS). UV–vis absorption spectroscopy was performed on a Scinco S‐3100 spectrophotometer. Steady‐state PL spectra were recorded using a Jasco FP‐8500 fluorometer. To examine the effect of solvent polarity on the emissive states of the DAN‐CDs, the solid samples were dissolved (0.05 mg/mL) in a series of aprotic (TOL, THF, ACN, DMF, and DMSO) and protic solvents (PeOH, BuOH, PrOH, MeOH, EtOH, and DW).

Femtosecond transient absorption (TA) spectra were acquired using a HELIOS TA spectrometer (Ultrafast Systems). Excitation pump pulses (330 nm) were generated by an optical parametric amplifier (TOPAS Prime, Light Conversion) driven by a 1 kHz Ti:sapphire amplifier (Legend Elite, Coherent; 790 nm, ∼30 fs). A visible white‐light continuum, generated via a nonlinear crystal, served as the broadband probe to monitor pump‐induced absorption changes across various time delays.

Time‐resolved PL (TRPL) decay dynamics were measured using Edinburgh Instruments FLS1000 (model FLS1000‐DD‐stm) and FS‐5 spectrometers equipped with pulsed laser diodes operating at excitation wavelengths of 375 and 475 nm. For the TRPL measurements, the DAN‐CDs and DAN‐CDs‐N_2_ were dispersed (0.05 mg/mL) in TOL and EtOH, respectively. Absolute PLQY measurements were performed on the FLS1000 spectrometer equipped with a 125‐mm‐diameter integrating sphere, with a 300 W steady‐state xenon lamp as the excitation source.

### 2.5 LED Fabrication and Characterization

ITO‐patterned glass substrates were sequentially cleaned by sonication in acetone and 2‐propanol for 15 min each. After drying, the substrates were treated with UV/ozone for 10 min to remove remaining organic residues and improve surface wettability. A buffer hole‐injection layer (Buf‐HIL), consisting of PEDOT:PSS and perfluorinated ionomer (PFI), was spin‐coated onto the cleaned ITO substrates, followed by thermal annealing at 150°C for 30 min. The emissive layer solutions were prepared by blending the DAN‐CDs with a TCTA:TPBI co‐host in anhydrous toluene. The TCTA:TPBI co‐host was fixed at a 1:1 weight ratio, while the DAN‐CD dopant concentration was systematically varied. The dopant concentration was controlled from 0 to 140 wt.% (defined as the mass percentage of DAN‐CDs relative to the total mass of the co‐host) in increments of 20 wt.%. The prepared blend solutions were deposited by spin‐coating at 1000 rpm for 60 s in a nitrogen‐filled glovebox. Afterward, TPBI (50 nm), LiF (1 nm), and Al (100 nm) were successively deposited by thermal evaporation under high‐vacuum conditions (base pressure 10^−7^ Torr). The EL characteristics of the resulting devices were measured using a Keithley 2450 source meter coupled with a Konica Minolta CS‐2000 spectroradiometer.

## 3 Results and Discussion

### 3.1 Synthesis Mechanism and Kinetics Simulation

**FIGURE 1:**
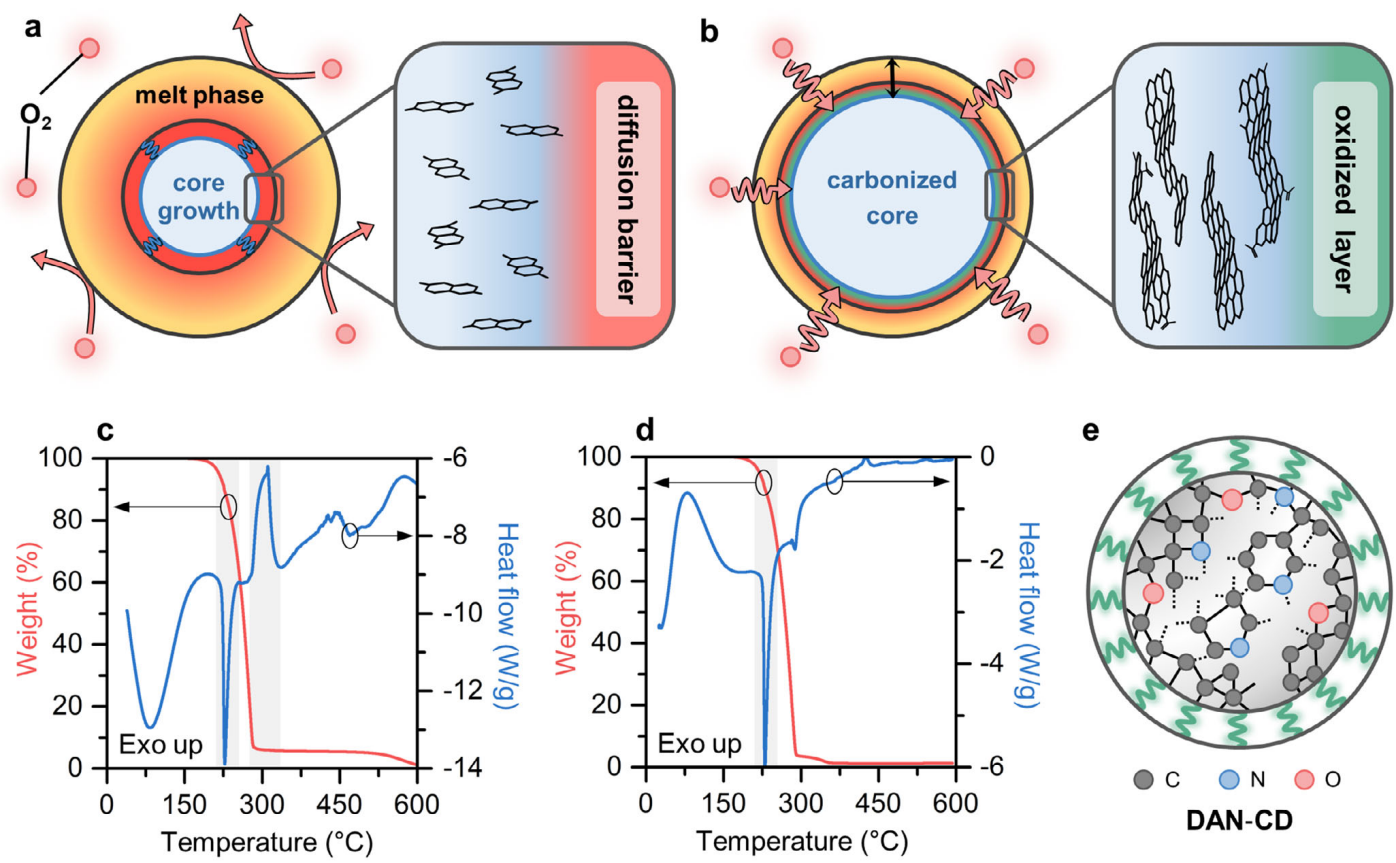
Proposed synthetic mechanism and structural evolution pathway for the formation of the DAN‐CDs via the solvent‐free, single‐precursor carbonization of DAN. (a) Growth of the conjugated carbonaceous core confined within an oxygen‐shielding precursor melt. (b) Subsequent oxygen‐mediated surface functionalization triggered by the attenuation of the diffusion barrier and the penetration of ambient oxygen. (c, d) TGA and DSC profiles of the DAN precursor under (c) oxygen and (d) nitrogen atmospheres. Exo denotes exothermic heat flow. (e) Schematic representation of the resultant DAN‐CDs.

The DAN‐CDs were synthesized through solvent‐free carbonization of DAN, utilizing it as a single molecular precursor without any additional additives (Figure 1 and Figure S1). Although the robust formation of an extensively conjugated carbonaceous core (a process typically requiring reductive or anoxic conditions) alongside the generation of oxygen‐rich surface functionalities presents an apparent mechanistic contradiction [32, 33], this paradox can be rationally elucidated through a spatiotemporally resolved precursor‐shielded growth mechanism [34].

During the early stages of the thermal reaction (Figure 1a and Figure S1a), the solid DAN precursor melts to form a dense, highly concentrated liquid matrix. This autogenic melt acts as a physical diffusion barrier, effectively shielding the nascent reaction centers from atmospheric oxygen [35, 36]. Confined within this transiently oxygen‐depleted microenvironment, DAN molecules undergo unhindered intermolecular amine‐mediated coupling to generate polycyclic aromatic intermediates [28, 37, 38, 39]. As the temperature increases, progressive deamination and further aromatization of these building blocks drive the formation of extensively conjugated, nitrogen‐doped domains. These thermally driven transformations primarily govern the development of the carbonaceous core, which is responsible for the core‐associated emissive states. As carbonization proceeds, the continuous consumption of the surrounding DAN monomers by the growing core progressively attenuates the protective precursor shield (Figure 1b and Figure S1b). The localized thinning of this diffusion barrier eventually allows ambient oxygen to penetrate and interact with the highly reactive peripheral edge sites of the carbon nanoseeds.

TGA and DSC measurements conducted under both oxygen (Figure 1c) and nitrogen (Figure 1d) atmospheres provide compelling empirical support for this staged transformation. Upon heating, a sharp endothermic peak is observed at 200°C–270°C in both atmospheres, precisely matching the melting point of the solid DAN precursor (∼220°C) and confirming the formation of the aforementioned autogenic melt. Concurrently, a mass loss occurs in this regime, indicative of thermal decomposition and the volatilization of amine groups [40]. The critical transition to the subsequent surface oxidation step is distinctly captured in the oxygen‐atmosphere DSC profile, where a strong exothermic peak emerges between 270°C and 340°C (Figure 1c). This peak, which is conspicuously absent under the nitrogen atmosphere (Figure 1d), represents the oxidation triggered by oxygen diffusion [41]. The partial oxidation of DAN‐derived intermediates, coupled with dehydration‐condensation and thermal rearrangement, facilitates the incorporation of oxygen‐containing surface functionalities. Consequently, the experimentally observed hydroxyl, carbonyl/carboxyl, and amide‐like groups strongly corroborate this progressive, oxygen‐mediated surface functionalization [42].

Crucially, the introduction of these sterically bulky and chemically stable oxidized moieties acts as an inherent passivation layer, leading to a self‐limiting intermolecular conjugation. This oxidative capping effectively terminates the further addition of DAN molecules to the core, successfully halting particle growth to yield well‐dispersed, uniformly sized CDs (Figure 1e). The stabilizing effect of this oxidative capping is further evidenced by the TGA profiles above 300°C. While the sample processed under a nitrogen atmosphere exhibits extremely low residual mass beyond 300°C due to unabated thermal decomposition, the sample processed under an oxygen atmosphere maintains a significantly higher mass retention over a broader range of 300°C–600°C. At elevated temperatures (e.g., 400°C), the attenuation of carboxylic acid‐related spectral features is consistent with the partial degradation of thermally unstable oxygenated groups, likely via decarboxylation [43].

Because our solvent‐free synthetic method eliminates the complex variables associated with liquid‐phase environments, its reaction kinetics are highly predictable and can be mathematically described. To formalize this spatiotemporally resolved dual‐pathway mechanism and quantitatively correlate it with the thermal analysis data, a kinetic model simulating the synthesis process was constructed (Figure S1c). The core hypothesis is that the local precursor concentration of the dense autogenic melt acts as a physical diffusion barrier that heavily inhibits premature oxidation. This dynamic can be modeled utilizing a concentration‐dependent shielding factor *f*_sd_ within a system of differential equations that tracks three key variables over time: the available precursor concentration [*P*], the radius of the carbonaceous core *R*, and the surface oxidation fraction θ. The growth rate of the carbonized core depends on both the fraction of active, unpassivated surface area (1 − θ) of the core and the available precursor pool ([*P*]):

$$\;\frac{dR}{dt}=k_{\mathrm{gr}}\;\left(1-\theta \right)\left[P\right]$$

where *k*_gr_ is the core growth rate constant, quantifying the kinetic ease of polycyclic aromatic condensation. Concurrently, the depletion of the precursor pool is directly driven by this core growth process:

$$\;\frac{d\left[P\right]}{dt}=-k_{\mathrm{gr}}\left(1-\theta \right)\left[P\right]$$

The critical transition from oxygen‐shielded core growth to oxygen‐mediated surface functionalization is captured by the oxidation rate equation. This rate depends on the remaining unoxidized active surface (1 − θ), the aforementioned precursor‐dependent shielding factor (*f*_sd_), and a constant atmospheric oxygen supply ([*O*]):

$$\;\frac{d\theta }{dt}=k_{\mathrm{ox}}\;\left(1-\theta \right)f_{\mathrm{sd}}\left[O\right]$$

where *k*_ox_ is the surface oxidation rate constant, quantifying the chemical affinity of the carbon core's peripheral edge sites for ambient oxygen. In our model, the local concentration of the precursor ([*P*]) dictates the physical thickness (*L*) of the melt phase surrounding the core; the characteristic time it takes for a gas to penetrate a physical barrier (its resistance to diffusion) is proportional to the square of the barrier's thickness according to Fick's second law of diffusion. Because diffusion resistance scales with *L* ^2^, and *L* is a function of the available precursor [*P*], the shielding capability naturally scales non‐linearly with [*P*]^2^:

$$\begin{array}{ccc} \;f_{\mathrm{sd}}=\frac{1}{1+\beta {\left[P\right]}^{2}}\; & \mathrm{and} & \;\frac{d\theta }{dt}=k_{\mathrm{ox}}\;\left(1-\theta \right)\frac{\left[O\right]}{1+\beta {\left[P\right]}^{2}} \end{array}$$

where β is an empirical shielding coefficient. To ensure the physical relevance of this model, β was not treated merely as a free mathematical fitting parameter. Instead, it was mathematically deduced by applying the final stabilized CD dimensions observed via TEM and DLS (mean radius ∼4 nm, discussed in Section 3.3) as a strict boundary condition. Because the completion of surface oxidation (θ→1) inherently terminates core growth (dR/dt→0), anchoring the simulated final radius to the experimental TEM data provides a precise physical value for β.

This geometrically constrained kinetic framework accurately captures the physical logic deduced from the macroscopic TGA and DSC profiles. During the initial stages of the synthesis, corresponding to the endothermic dense autogenic melt regime (200°C–270°C), the unreacted precursor concentration ([*P*]) is at its peak. Consequently, the [*P*]^2^ is exceptionally large, rendering the overall shielding factor mathematically close to 0. Under these conditions, *d*θ/*dt* ≈ 0, meaning oxidation is effectively blocked. This allows for unhindered core expansion (*dR*/*dt* > 0) driven by precursor consumption, which perfectly correlates with the initial mass loss and the absence of exothermic oxidation features in this temperature regime.

However, as the reaction progresses and precursor molecules are continuously consumed by the growing core (*d*[*P*]/*dt* < 0), the protective melt layer progressively thins. Once [*P*] depletes below a critical threshold, the denominator of the shielding factor decreases, causing the factor to sharply approach 1. This structural exposure to ambient oxygen triggers an abrupt and massive spike in the oxidation rate (*d*θ/*dt*). This mathematically formulated oxidation spike precisely mirrors the sudden emergence of the strong exothermic peak uniquely observed in the oxygen‐atmosphere DSC curve between 270°C and 340°C.

Finally, as the rapid influx of oxygen creates functional groups across the core surface, θ→1. This forces the active surface term (1 − θ) to zero, which simultaneously drives both the core growth (*dR*/*dt*) and the oxidation rate (*d*θ/*dt*) strictly to zero. This mathematical limit rigorously validates the self‐limiting “oxidative capping” mechanism. It explicitly demonstrates how surface oxidation acts as an inherent passivation layer that terminates further structural expansion, thereby yielding uniformly sized DAN‐CDs and explaining the significantly higher mass retention plateau observed in the oxygen‐atmosphere TGA profile above 300°C. The structural transformations inherent to this spatiotemporally regulated dual‐pathway mechanism are further elucidated through the following temperature‐dependent FTIR and NMR investigations.

### 3.2 Chemical Characterization

**FIGURE 2:**
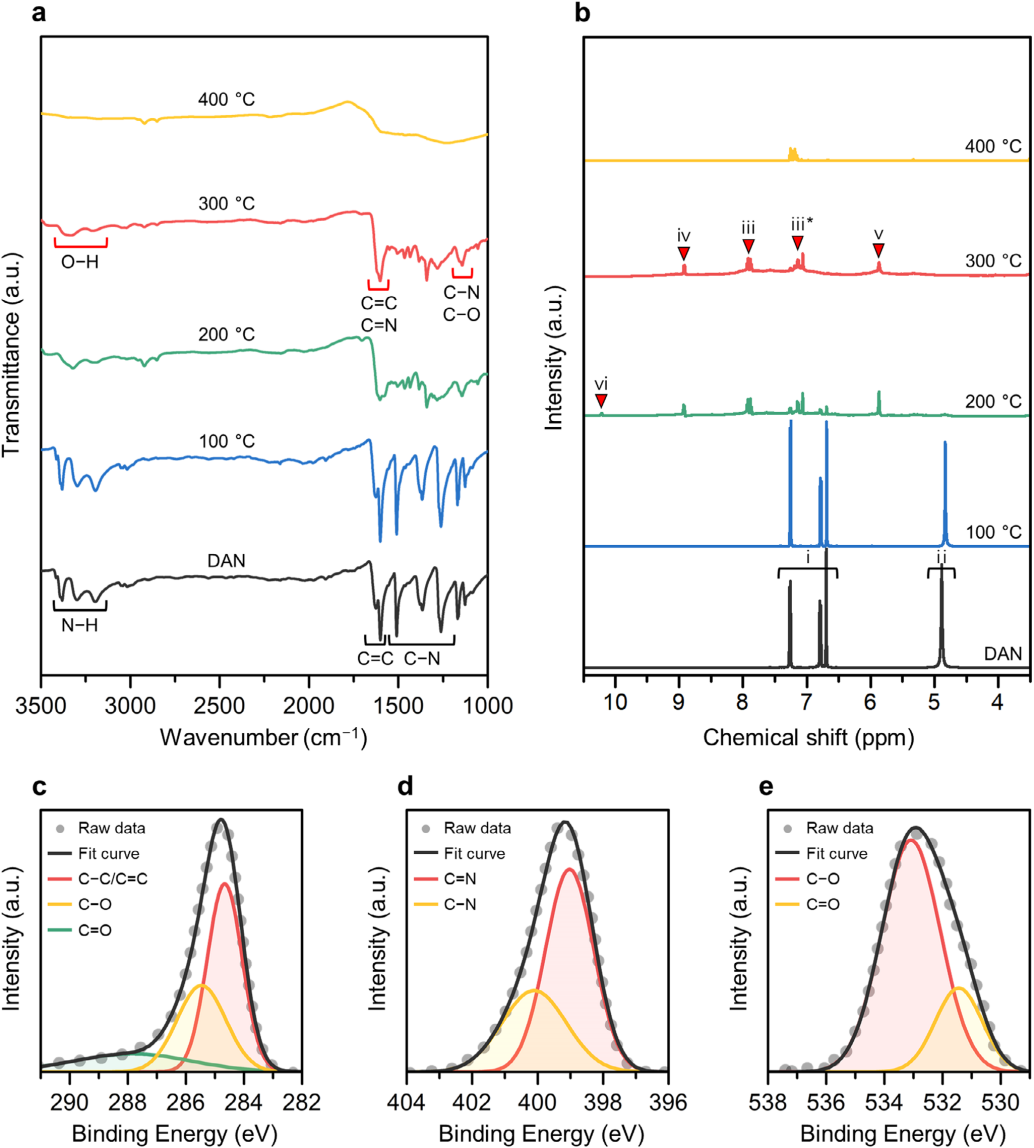
(a) FTIR and (b) ^1^H NMR spectra of the pristine DAN precursor and the DAN‐CDs synthesized at varying carbonization temperatures (100°C, 200°C, 300°C, and 400°C). High‐resolution XPS spectra of the (c) C 1s, (d) N 1s, and (e) O 1s regions for the optimized DAN‐CDs prepared at 300°C.

FTIR spectroscopy was utilized to monitor the temperature‐dependent evolution of functional groups in the DAN‐CDs (Figure 2a). The pristine DAN precursor exhibited distinct N─H stretching of primary amines at 3352–3425 cm^−1^ (centered at 3382 cm^−1^), along with additional N─H stretching features at 3240–3340 cm^−1^ and 3120–3240 cm^−1^. Corresponding absorption signals at 1301–1407 cm^−1^ and 1187–1310 cm^−1^ were attributed to C─N stretching vibrations of aromatic amine moieties [44]. The aromatic framework of DAN was further evidenced by a prominent band at 1528–1657 cm^−1^, corresponding to aromatic C═C stretching [45]. These features were largely preserved in the sample treated at 100°C, indicating that the precursor structure remained predominantly intact and that significant carbonization had not yet initiated.

In contrast, distinct spectral transformations were observed for the DAN‐CDs prepared at ≥ 200°C. The characteristic N─H and C─N precursor bands were markedly attenuated, confirming the substantial consumption and structural transformation of amine groups during thermal condensation. Concomitantly, a broad band emerged at 3263–3404 cm^−1^, which is consistent with O─H stretching and suggests the formation of hydroxyl functionalities [46]. Emergent absorption signals were also observed at 1536–1660 cm^−1^ and 1102–1185 cm^−1^. The former is attributed to aromatic C = C and/or conjugated C = N vibrations, likely including contributions from newly formed oxygenated or heteroatom‐doped domains [47]. The latter is consistent with C─O stretching and/or C─N stretching within oxygenated or amide‐like environments [48]. These bands became significantly more pronounced as the carbonization temperature increased from 200°C to 300°C, confirming the progressive formation of oxygen‐containing and amide‐like functionalities during oxidative carbonization.

For comparison, the FTIR spectrum of the inert‐synthesized control sample (DAN‐CDs‐N_2_) exhibited distinctly different structural characteristics (Figure S2a). In the 3120–3425 cm^−1^ region, weak residual signals were observed, indicating the presence of unreacted or partially condensed amine‐like species similar to those in the DAN precursor. However, unlike the air‐synthesized DAN‐CDs, the DAN‐CDs‐N_2_ sample lacked the broad O─H stretching band centered at 3319 cm^−1^ and the distinct C─O/amide C─N stretching band in the 1102–1185 cm^−1^ region. Nevertheless, the conjugated C═C/C═N band at 1536–1660 cm^−1^ remained present, confirming that aromatic condensation successfully occurs even in the absence of oxygen.

^1^H NMR spectroscopy was subsequently employed to trace the chemical evolution of the DAN‐CDs across different carbonization temperatures (Figure 2b). The DAN precursor exhibited characteristic aromatic proton resonances in the range of 6.6–7.3 ppm (i), corresponding to the naphthalene framework, along with well‐resolved signals at 7.25–7.27 ppm (H‐1 or H‐5), 6.78–6.80 ppm (H‐3 or H‐7), and ∼6.7 ppm (H‐4 or H‐8). Additionally, a resonance at 4.89 ppm (ii) was assigned to primary amine protons (─NH_2_) [49]. Similar to the FTIR results, these spectral features were entirely preserved at 100°C, confirming the thermal stability of the DAN precursor at low temperatures. However, upon carbonization at 200°C, a pronounced decrease in the primary amine signal (ii) was observed, accompanied by the emergence of new aromatic resonances between 7.0 and 8.0 ppm (iii and iii^*^). Signals near 7.86–7.88 ppm and 7.91–7.94 ppm became discernible and were assigned to strongly deshielded aromatic environments, likely corresponding to newly formed nitrogen‐doped aromatic or heteroaromatic moieties [50]. Furthermore, emergent signals at ∼8.9 ppm (iv), 5.9 ppm (v), and ∼10.2 ppm (vi) were detected, which are consistent with amide‐like, hydroxyl‐type, and carboxylic acid protons, respectively [51, 52, 53]. This observation strongly corroborates the formation of oxygen‐rich surface functional groups during intermediate‐temperature oxidative carbonization.

In the optimized sample carbonized at 300°C, the deshielded aromatic resonances (iii and iii^*^) and the amide‐like signal (iv) persisted, while the precursor amine proton signal (ii) completely vanished, confirming the exhaustive consumption of primary amines. Notably, the carboxylic acid proton signal (vi) was no longer detected at 300°C, supporting the FTIR observation of thermal decarboxylation. Finally, at 400°C, all proton signals associated with surface functional groups (iv–vi) disappeared, leaving only weak residual aromatic signatures. This points to advanced carbonization and extensive structural cross‐linking, yielding a highly condensed graphitic framework stripped of passivating functional groups [54].

Correspondingly, the ^1^H NMR spectrum of DAN‐CDs‐N_2_ (Figure S2b) presented a different chemical profile. The DAN‐CDs‐N_2_ sample exhibited broad resonances spanning 6.5–8.5 ppm, attributable to heteroaromatic protons in highly varied conjugated environments, alongside a residual amine‐like proton signal in the 4.4–5.1 ppm region. Critically, the signature oxygen‐related surface functionalities observed in the air‐synthesized DAN‐CDs, such as the amide‐like proton (∼8.9 ppm, iv) and the hydroxyl‐type proton (∼5.87 ppm, v), were entirely absent. The lack of these specific features demonstrates that an inert carbonization atmosphere suppresses the formation of oxygenated surface states, whereas the robust aromatic resonances confirm that the thermal generation of a nitrogen‐doped core remains unimpeded. Overall, the combined FTIR and ^1^H NMR analyses explicitly verify that while nitrogen‐containing conjugated aromatic domains form in both materials, oxygen‐containing surface functionalities uniquely develop only under oxidative conditions.

To conclusively determine the elemental composition and chemical bonding environments implied by the FTIR and NMR analyses, XPS was performed on the optimized DAN‐CDs prepared at 300°C (Figure 2c–e). The high‐resolution C 1s spectrum (Figure 2c) was deconvoluted into three distinct components. The dominant peak centered at 284.6 eV was assigned to C─C/C═C bonds, confirming the presence of an sp^2^‐enriched carbon framework. The component at 285.5 eV was attributed to C─O bonds, verifying the integration of oxygen‐containing surface functionalities. A higher‐binding‐energy peak at 287.8 eV was assigned to carbonyl‐containing species (C═O), encompassing oxidized carbon and amide‐like functionalities [55]. The N 1s spectrum (Figure 2d) displayed two characteristic peaks. The primary peak at 399.0 eV was assigned to C═N bonds, typically associated with pyridinic nitrogen embedded within conjugated aromatic domains [56]. These pyridinic nitrogen species are critical to the photophysics of the core, as their lone‐pair electrons participate in the conjugated *π*‐system and directly modulate the internal electronic band structure [57]. The secondary peak at 400.1 eV was attributed to C─N bonds, representing pyrrolic or amide‐type nitrogen species [55]. The O 1s spectrum (Figure 2e) was resolved into two components: a peak at 531.4 eV corresponding to carbonyl oxygen (C═O) and a higher‐binding‐energy peak at 533.1 eV assigned to C─O bonds (e.g., hydroxyl groups) [58]. These highly electronegative oxygen functionalities introduce localized, electron‐withdrawing edge states [37, 59]. The quantitative XPS results are in excellent agreement with the FTIR and NMR data, collectively verifying that the DAN‐CDs possess a highly condensed, sp^2^‐enriched, nitrogen‐doped carbon core uniformly decorated with passivating oxygenated surface functionalities.

Finally, to explicitly verify the dynamic temporal timelines predicted by our kinetic model, specifically the initial suppression of oxidation and the subsequent sudden onset of oxidative capping, we conducted systematically segmented control experiments tracking the structural evolution of the intermediates at 200°C, 250°C, and 300°C over various time intervals (Figures S3–S7 and Table S2). During the initial heating stages (200°C–250°C), the XPS survey and high‐resolution O 1s spectra showed negligible structural oxygen content, while the N 1s spectra and FTIR confirmed the strict persistence of primary amines. This provides direct structural evidence that the dense autogenic melt acts as a highly effective physical barrier, completely blocking ambient oxygen and allowing unhindered initial core formation. As the reaction progresses at 300°C (up to 1 h), the continuous consumption of the precursor thins this barrier, marked by the emergence of pyrrolic and pyridinic nitrogen and the attenuation of primary amines. Once the barrier depletes below a critical threshold (3–6 h), structural exposure to oxygen triggers the massive oxidation phase mathematically predicted by our model. This is physically evidenced by a radical increase in the oxygen atomic percentage (from 4.14% at 1 h to 15.95% at 6 h) and a dramatic surge in C═O and C─O species in the high‐resolution XPS spectra. Ultimately, under prolonged thermal treatment (24 h), partial thermal degradation of unstable carboxyl groups leads to a stabilized oxygen ratio (9.05%) and the highly passivated, thermodynamically stable CD core depicted in Figure 2. This sequential, time‐resolved structural evolution supports the spatiotemporal progression from oxygen‐shielded core growth to self‐limiting oxidative capping.

### 3.3 Morphological and Structural Characterization

To visually corroborate the spatiotemporal dynamic model established by our chemical analyses, we systematically tracked the morphological evolution of the DAN‐CDs across different reaction stages using DLS and TEM (Figures S8–S12). At lower temperatures (200°C–250°C), DLS revealed massive structures (mean diameters > 1300 nm), and wide‐field TEM images displayed predominantly amorphous, macroscopic aggregates. This provides direct physical evidence of the autogenic melt phase, where precursor molecules fuse into a dense liquid matrix. Upon reaching 300°C, distinct spherical particles began to segregate from this bulk phase. As the reaction proceeded at 300°C (from 0 to 24 h), the mean particle size visibly and continuously decreased, reflecting the progressive consumption of the precursor melt. DLS measurements showed the mean size narrowing from 40.4 nm (0 h) down to 7.5 nm (3 h). Correspondingly, HR‐TEM confirmed the emergence of distinct crystalline lattice fringes within these shrinking domains, indicating the maturation of the carbonaceous core as surface oxidation stabilizes the particles against further aggregation. Ultimately, this self‐limiting process yields the highly uniform, optimized DAN‐CDs.

**FIGURE 3:**
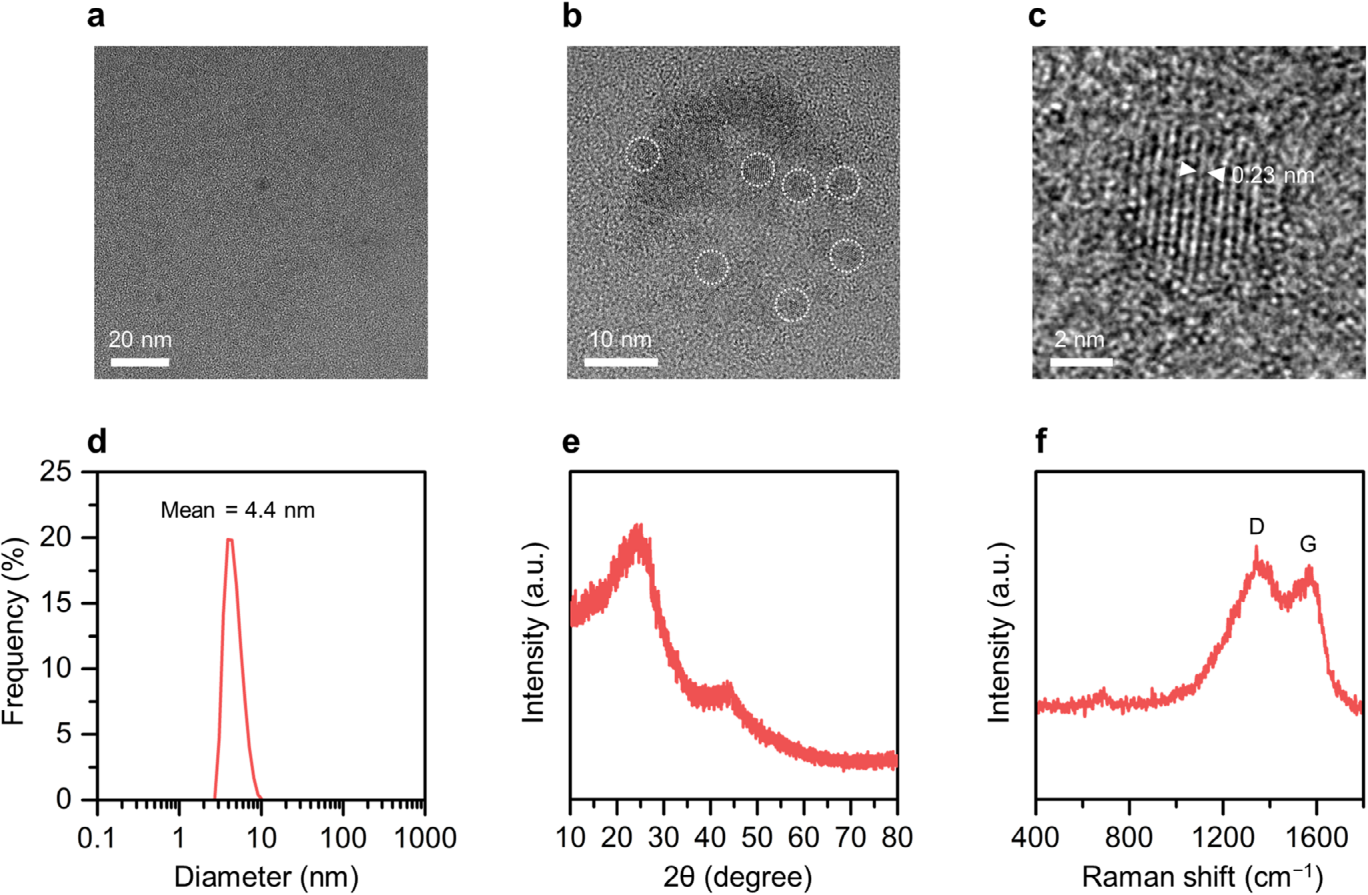
Morphological and structural characterization of the optimized DAN‐CDs. (a, b) TEM images at different magnifications. (c) HR‐TEM image displaying localized graphitic lattice fringes. (d) Hydrodynamic size distribution obtained via DLS. (e) XRD pattern. (f) Raman spectrum with the D and G bands explicitly indicated.

As shown in the TEM and DLS analyses of the final stabilized product (Figure 3a–d), the synthesized CDs are well‐dispersed, quasi‐spherical nanoparticles with diameters predominantly ranging from 4 to 8 nm. HR‐TEM further elucidated the internal structure, displaying partially ordered nanoscale domains with distinct lattice fringes exhibiting an interplanar spacing of 0.23 nm (Figure 3c). This spacing correlates well with the (100) in‐plane lattice facet of graphitic carbon. The limited spatial continuity of these fringes indicates that the DAN‐CDs predominantly consist of a disordered carbon matrix embedded with localized, short‐range sp^2^‐hybridized graphitic domains [60]. Corroborating the TEM observations, DLS measurements yielded a narrow hydrodynamic size distribution with a mean diameter of 4.4 nm (Figure 3d), confirming the highly uniform nanoscale dimensions of the DAN‐CDs in the solution state.

XRD analysis was performed to assess the long‐range crystallinity and the degree of graphitization of the DAN‐CDs (Figure 3e). The XRD pattern displayed a single, highly broadened diffraction peak centered at 2θ ≈ 24°, which is assigned to the (002) interlayer reflection of disordered, turbostratic‐like carbon. The pronounced broadness of this peak, coupled with the absence of sharp crystalline diffraction features, indicates a lack of extended graphitic stacking within the carbon framework [60, 61]. These structural characteristics suggest that the DAN‐CDs possess a largely amorphous carbon architecture punctuated by limited graphitic ordering, precisely mirroring the localized sp^2^ domains observed via HR‐TEM.

Raman spectroscopy was utilized to further probe the structural disorder and defect density within the carbon framework of the DAN‐CDs (Figure 3f). The spectrum exhibited two prominent carbon‐related features: the D‐band centered at 1358 cm^−1^ and the G‐band at 1567 cm^−1^. The G‐band arises from the in‐plane vibrational mode of sp^2^‐hybridized carbon atoms within ordered graphitic domains, whereas the D‐band originates from defects, edge states, and structural disorder within the lattice [62, 63]. The integrated intensity ratio of the D‐band to the G‐band (I_D_/I_G_) was calculated to be 1.01. This high ratio quantitatively corroborates TEM and XRD findings, pointing to a high density of surface/edge defects and a predominantly disordered, turbostratic carbon framework enriched with heteroatom functionalization. Overall, the integrated TEM, DLS, XRD, and Raman data consistently demonstrate that the DAN‐CDs are composed of localized nanoscale sp^2^ domains embedded within a highly defective, functionalized carbon matrix.

### 3.4 Optical Properties and Excited‐State Dynamics

**FIGURE 4:**
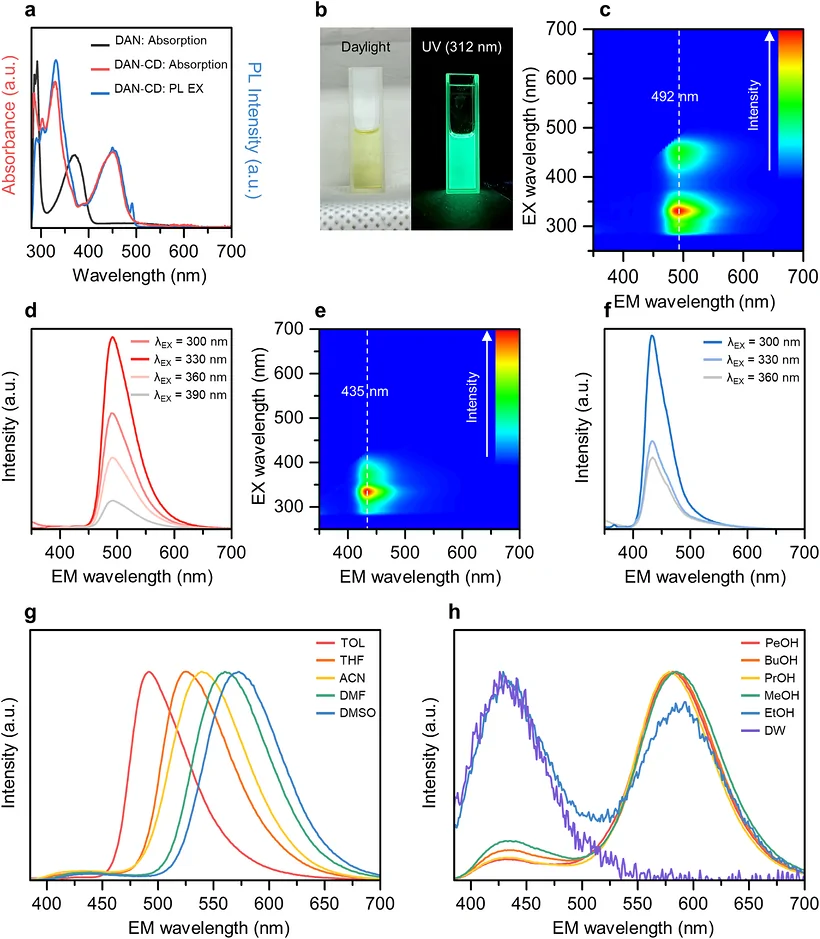
Steady‐state optical properties. (a) Normalized UV–vis absorption spectra of the pristine DAN precursor and the DAN‐CDs, alongside the PL excitation spectrum of the DAN‐CDs (λ_EM_ = 492 nm). (b) Photographs of the DAN‐CDs dispersed in TOL (1 mg/mL) under ambient daylight (left) and 312 nm UV irradiation (right). (c) PL emission contour map and (d) corresponding PL emission spectra (λ_EX_ = 300, 330, 360, and 390 nm) of the DAN‐CDs. (e) PL emission contour map and (f) corresponding PL emission spectra (λ_EX_ = 300, 330, and 360 nm) of the DAN‐CDs‐N_2_. (g, h) Solvatochromic PL emission spectra of the DAN‐CDs dispersed in (g) aprotic and (h) protic solvents. EX and EM denote excitation and emission, respectively.

UV–vis absorption spectroscopy was performed to investigate the electronic transitions of the pristine DAN precursor and the optimized DAN‐CDs (Figure 4a). The DAN precursor exhibited two characteristic absorption regions: a high‐energy band in the range of 270–300 nm, attributed to the *π*–*π* ^*^ transitions of the naphthalene aromatic framework, and a broader feature between 300 and 400 nm, associated with the n‐*π* ^*^ transitions involving lone‐pair electrons of the primary amine functionalities [64, 65]. Following carbonization at 300°C, the resultant DAN‐CDs displayed three distinguishable absorption features: a band at 270–300 nm, a distinct peak at ∼330 nm, and a broad absorption tail extending from 400 to 500 nm. The high‐energy band (270–300 nm) is assigned to the *π*–*π* ^*^ transitions within the newly formed, sp^2^‐enriched polyaromatic carbon core. The peak near 330 nm is attributed to the n‐*π* ^*^ transitions associated with the nitrogen‐doped conjugated carbon framework [66, 67]. Specifically, the lone‐pair electrons of the incorporated pyridinic and pyrrolic nitrogen heteroatoms strongly hybridize with the polyaromatic *π*‐network, effectively narrowing the intrinsic HOMO‐LUMO gap of the core [57, 68]. The broad low‐energy absorption tail extending from 400 to 500 nm is linked to surface‐related electronic states generated during oxidative carbonization. The incorporation of strongly electronegative oxygen moieties (e.g., carbonyl and carboxyl groups) at the peripheral edge sites breaks the structural symmetry and introduces localized, mid‐gap defect states [69, 70]. The PL excitation spectrum, monitored at the primary emission wavelength of 492 nm, is in excellent agreement with the absorption spectrum. It strongly overlaps with the 330 and 400–500 nm absorption features while showing minimal contribution from the high‐energy *π*–*π* ^*^ core band. This observation clearly indicates that the dominant emissive pathways are intimately associated with intermediate heteroatom‐related and surface‐defect electronic states.

Photographs of the DAN‐CDs demonstrate a pale‐yellow color under ambient daylight and an intensely bright green emission under 312 nm UV irradiation (Figure 4b). The PL emission contour map (Figure 4c) and its corresponding spectra (Figure 4d) recorded across various excitation wavelengths reveal a pronounced, excitation‐independent emission profile centered at 492 nm, achieving maximum intensity at an excitation wavelength of 330 nm. Notably, this emission exhibits a narrow FWHM (< 60 nm) compared to conventional CDs [71, 72, 73], indicating a highly uniform and well‐defined excited‐state relaxation pathway. This confirms that the solvent‐free synthetic approach effectively restricts the random formation of diverse, non‐radiative defect motifs. Along with this significantly broadened excitation responsiveness, the DAN‐CDs demonstrate a substantial bathochromic (red) shift of ∼75 nm compared to the pristine DAN precursor, which exhibits a broad blue emission centered at ∼420 nm (Figure S13). This pronounced red shift physically manifests from the spatial extension of the *π*‐conjugated electron system and the energetic stabilization provided by the newly formed, electron‐withdrawing oxygen surface functionalities, which act as sub‐band‐gap radiative traps [74, 75].

To explicitly isolate the photophysical roles of the incorporated nitrogen and oxygen, the DAN precursor was carbonized under an inert nitrogen atmosphere to yield the DAN‐CDs‐N_2_ control sample. Unlike the air‐synthesized DAN‐CDs, the broad, oxygen‐related low‐energy absorption tail (400–500 nm) was notably absent in the absorption spectrum of the DAN‐CDs‐N_2_ (Figure S14). Furthermore, their PL contour map (Figure 4e) and corresponding spectra (Figure 4f) reveal an excitation‐independent emission centered at a higher energy (435 nm). This pronounced contrast provides critical mechanistic insight by demonstrating that the 435 nm emission corresponds to an intrinsic, nitrogen‐doped core state present in both samples. Because the core emissive centers are structurally embedded within the rigid, hydrophobic sp^2^ backbone, they establish the primary optical bandgap without the influence of oxygen. Conversely, the dominant lower‐energy emissive state (492 nm) observed exclusively in the DAN‐CDs is directly engendered by the highly polar, oxygen‐mediated functionalization during aerobic carbonization, generating lower‐energy traps that efficiently capture excited electrons [76, 77].

To gain deeper structural insight into the electronic nature of these dual emissive states, the solvatochromic PL behavior of the DAN‐CDs was investigated (Figure 4g,h). In aprotic solvents, the dominant long‐wavelength emission band (492 nm in TOL) exhibited positive solvatochromism, gradually red‐shifting by ∼100 nm as solvent polarity increased (TOL→THF→ACN→DMF→DMSO). This pronounced positive solvatochromism is fundamentally tied to an intramolecular charge transfer (ICT) mechanism from the electron‐rich nitrogen‐doped core to the electron‐withdrawing oxygenated surface groups [78]. The high density of polar oxygen moieties creates a large excited‐state dipole moment across the CD surface. As solvent polarity increases, these surface states are preferentially stabilized via dielectric relaxation and dipole‐dipole interactions, markedly lowering the emission energy [79]. Interestingly, the weak secondary emission feature near 435 nm exhibited negligible spectral variation. This confirms its origin from the deeply embedded, sterically shielded nitrogen‐doped sp^2^ core, which lacks the strong directional dipole necessary for extensive solvent coupling [80]. In protic solvents (e.g., PeOH, BuOH, PrOH, MeOH, EtOH, and DW), strong intermolecular hydrogen‐bonding interactions with the oxygenated surface functionalities provided profound excited‐state stabilization, driving a significant red shift of the long‐wavelength emission to ∼585 nm. Simultaneously, the highly hydrophobic nature of the DAN‐CDs induced physical aggregation in these highly polar media. This aggregation partially quenched the red‐shifted 585 nm surface emission via aggregation‐caused quenching (ACQ) [81, 82], subsequently allowing the unperturbed, solvent‐insensitive 435 nm core emission to appear increasingly pronounced.

Crucially, this solvent‐induced aggregation is driven entirely by physical hydrophobic interactions and is fully reversible. As demonstrated through solvent‐exchange experiments (Figure S15), recovering the aggregated DAN‐CDs from protic solvents (EtOH and DW) and redispersing them into an aprotic solvent (TOL) results in the complete disassembly of the clusters. DLS measurements confirm that the heavily aggregated clusters (with mean diameters of 60.4 nm in EtOH and 285.4 nm in DW) revert to their highly uniform, monomeric nanoscale dimensions (∼3.9–4.6 nm). Consequently, their original high‐PLQY green emission spectra are completely restored. This robust reversibility confirms that the highly polar surface functional groups of the DAN‐CDs cause transient, solvent‐dependent assembly rather than permanent aggregation, ensuring they remain suited for the standard solution‐processing in aprotic solvents typically required for LED emissive layer fabrication. Collectively, these solvatochromic results definitively assign the 435 nm emission to the structurally shielded nitrogen‐doped core, and the highly tunable 492 nm emission to an exposed, oxygen‐mediated surface state.

**FIGURE 5:**
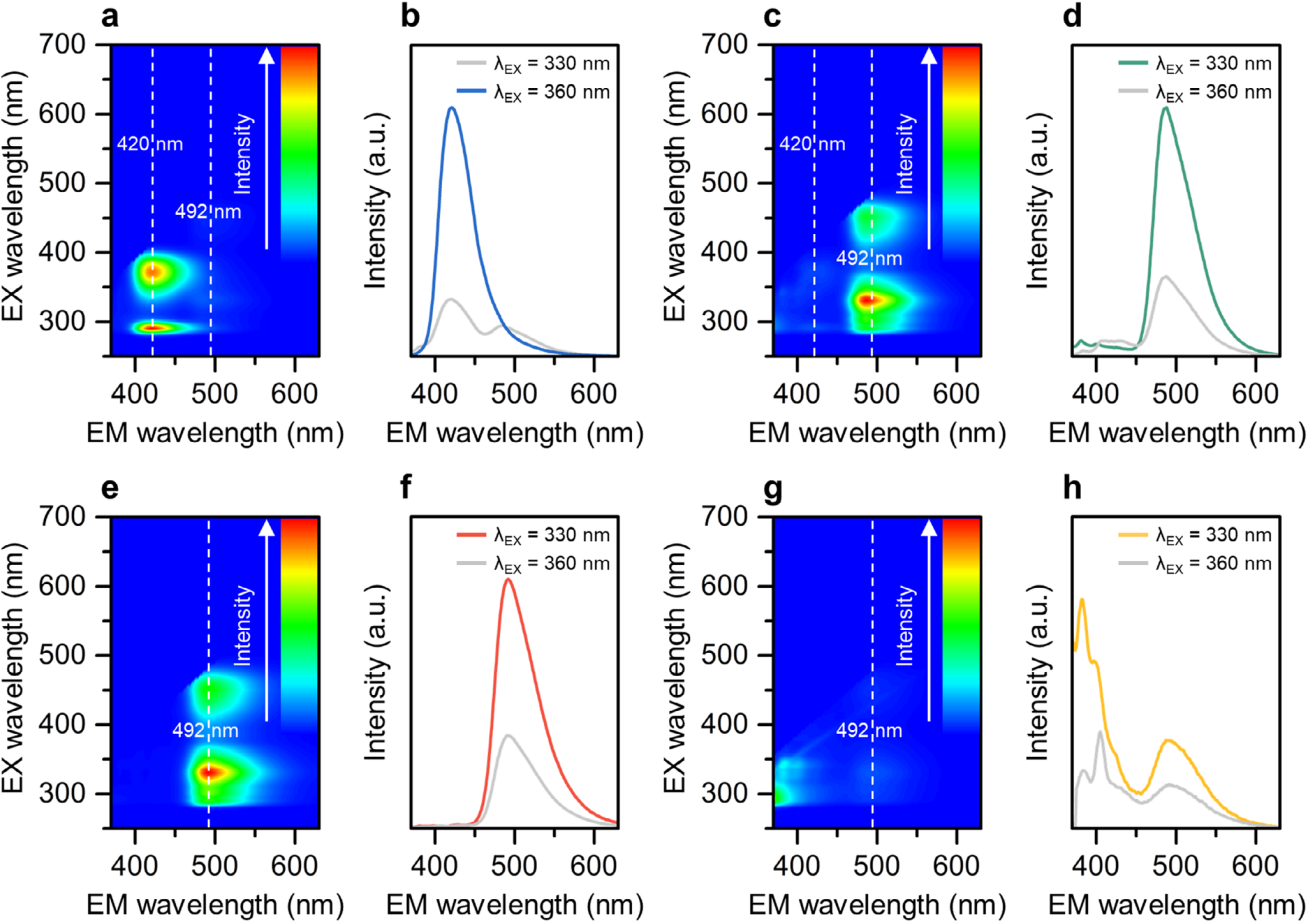
Temperature‐dependent optical properties. (a, c, e, g) PL emission contour maps and (b, d, f, h) corresponding PL emission spectra of the DAN‐CDs synthesized at varying carbonization temperatures: (a, b) 100°C, (c, d) 200°C, (e, f) 300°C, and (g, h) 400°C.

The temperature‐dependent evolution of these emissive states further confirms this mechanism (Figure 5 and Figure S16). The sample treated at 100°C largely retained the precursor‐like emission near 420 nm, aligning with the minimal structural transformation confirmed by the previous FTIR and ^1^H NMR analyses. At 200°C, the precursor‐associated emission diminished, and a new emission band emerged near 492 nm, signifying the initial generation of oxygen‐passivated carbonized domains. The optimized 300°C sample exhibited emission at this identical wavelength but with drastically enhanced intensity, indicating the optimal thermodynamic proliferation of highly luminescent conjugated domains and passivated surface states. Conversely, carbonization at 400°C induced optical quenching, likely due to excessive structural cross‐linking and thermal decarboxylation, which degrades passivating oxygen functionalities and introduces non‐radiative defect centers [83].

Having established 300°C as the optimal carbonization temperature, we sought to further validate the rational geometric design behind the selection of 2,6‐DAN as the singular molecular precursor. To this end, solvent‐free carbonization was performed using its positional isomers (1,5‐, 1,8‐, and 2,3‐DAN) under identical thermal conditions. As evidenced by the UV–vis absorption spectra (Figure S17), the optimized 2,6‐DAN‐CDs displayed distinct, well‐resolved absorption bands indicative of a structurally uniform, extended *π*‐conjugated network. In contrast, the CDs derived from the asymmetric or sterically hindered isomers exhibited anomalous, significantly broadened (1,5‐ and 1,8‐DAN‐CDs), or nearly featureless, continuously decaying (2,3‐DAN‐CDs) absorption profiles, directly reflecting massive structural heterogeneity and a collective failure to assemble a coherent optical bandgap.

This ground‐state structural disparity was mirrored in their emissive behaviors (Figure S18). While the optimized 2,6‐DAN‐CDs displayed a singular, well‐defined, and excitation‐independent emission centered at 492 nm with a narrow FWHM of 61.6 nm, the suboptimal isomers yielded highly heterogeneous, multi‐band optical profiles. Specifically, the carbonization of 1,5‐, 1,8‐, and 2,3‐DAN resulted in PL spectra dominated by strong residual high‐energy emissions (< 400 nm), indicative of unreacted precursors or minimally coupled oligomers. Concurrently, their carbonization‐induced emissive states were severely quenched, broadened, and red‐shifted to 532, 560, and 534 nm, respectively, accompanied by massive FWHM expansions to 85.4, 143.5, and 136.0 nm.

This optical contrast is fundamentally rooted in molecular topology. The linear, symmetrically opposed positioning of the primary amines in 2,6‐DAN provides an ideal geometric building block. It minimizes steric hindrance and facilitates a highly ordered, continuous head‐to‐tail polyaromatic condensation, allowing for the exhaustive structural conversion of the precursor into a uniform conjugated network. In contrast, isomers with adjacent amine groups (e.g., 1,8‐ and 2,3‐DAN) suffer from severe steric strain, which drives premature, highly localized intramolecular cyclization. This chain‐terminating behavior restricts extensive polymerization, physically trapping the materials as a chaotic mixture of small fluorescent oligomers (evidenced by the persistent high‐energy blue emissions) and defect‐ridden amorphous clusters. Similarly, the structurally offset amines in 1,5‐DAN induce an asymmetric, kinked polymerization, generating a highly strained, heterogeneous matrix populated with non‐radiative defect traps. Consequently, the symmetric linear extension uniquely afforded by 2,6‐DAN is structurally indispensable for assembling the robust, highly efficient emissive centers that yield the narrow FHWM and near‐unity PLQY.

**FIGURE 6:**
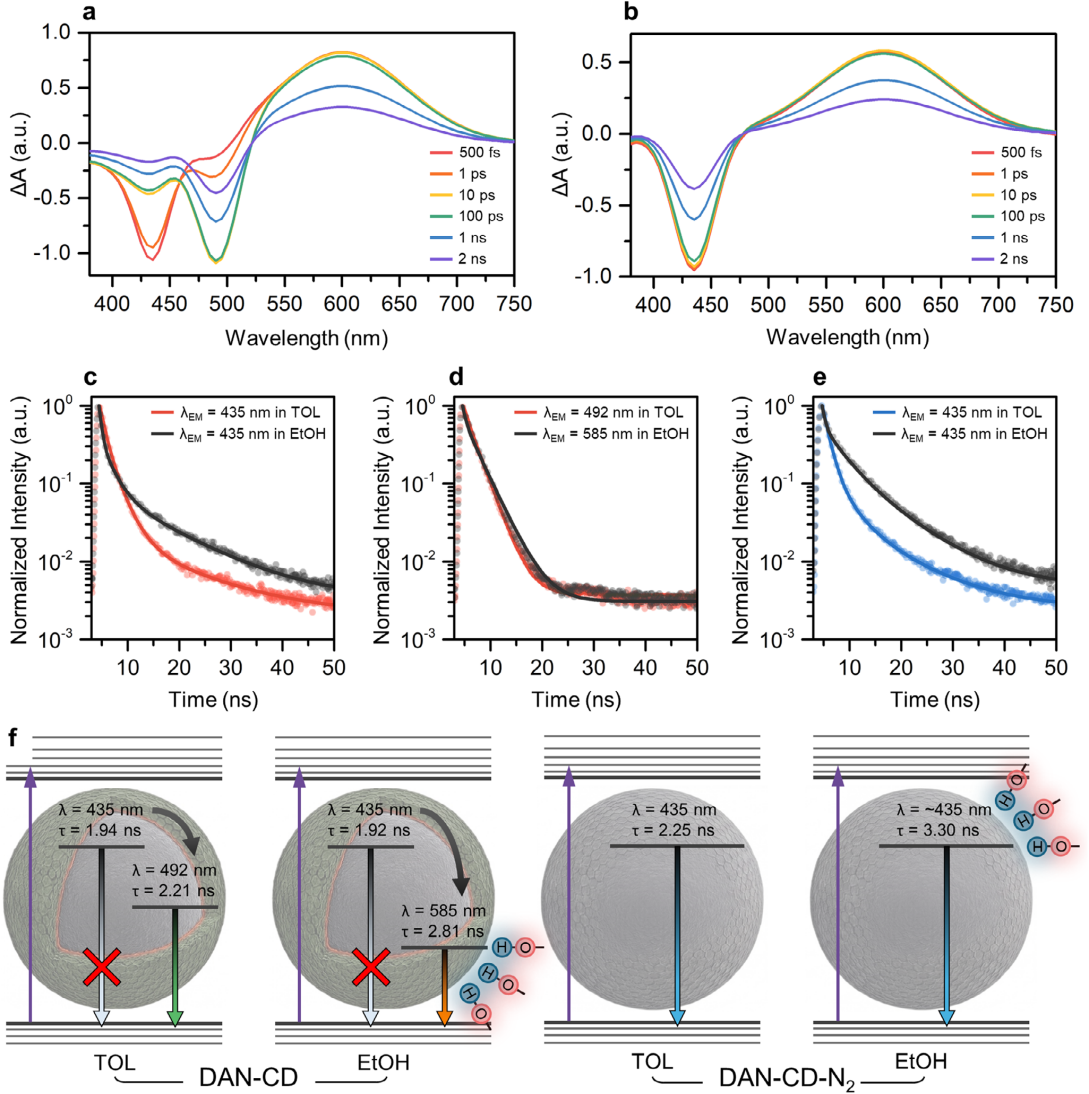
TA spectra of the (a) optimized DAN‐CDs and (b) DAN‐CD‐N_2_ at various probe delays (λ_EX_ = 330 nm). TRPL decay curves recorded in TOL and EtOH under 375 nm pulsed laser excitation. The DAN‐CDs were monitored at (c) the core‐associated emission state (λ_EM_ = 435 nm) and (d) the solvent‐dependent, surface‐associated emission state (λ_EM_ = 492 nm in TOL and 585 nm in EtOH). (e) The DAN‐CDs‐N_2_ monitored at the core emission state (λ_EM_ = 435 nm). (f) Schematic energy‐level diagram illustrating the proposed solvent‐dependent electronic transitions and relaxation dynamics associated with the core and surface states.

To directly observe the ultrafast charge transfer dynamics bridging these dual emissive centers, femtosecond TA spectroscopy was performed (Figure 6a,b). Upon 330 nm pump excitation, the TA spectrum of the DAN‐CDs reveals a broad ground‐state bleaching (GSB) tail (400–480 nm) and two distinct negative stimulated emission (SE) bands corresponding to the core (∼435 nm) and surface (∼495 nm) states (Figure 6a). At early probe delays (500 fs to 1 ps), the core state is instantly populated. Crucially, within the ultrafast domain (1–10 ps), the 435 nm core SE band rapidly decays while the 495 nm surface SE band simultaneously deepens. This ultrafast spectral evolution provides direct evidence of excitons efficiently funneling down the energy gradient from the internal light‐harvesting core into the terminal surface traps.

Conversely, the TA spectrum of the inert‐synthesized DAN‐CD‐N_2_ control lacks the broad 400–500 nm GSB tail, consistent with its narrowed steady‐state absorption spectrum (Figure S14), confirming the absence of oxygen surface defect states. Consequently, only a single, static core SE band at ∼435 nm emerges (Figure 6b). Because it lacks these electronegative functionalities, no ultrafast picosecond spectral shift occurs, confirming that excitons are instantly generated and remain trapped in the core.

To elucidate the specific excited‐state charge dynamics governing this dual‐emission mechanism, TRPL measurements were conducted (Figure 6c–e and Tables 1 and 2). All PL decay curves were fitted using a bi‐exponential decay function, yielding distinct short (τ_1_) and long (τ_2_) lifetime components, indicative of multiple relaxation channels within the emissive manifold. For the DAN‐CDs excited at 375 nm (Figure 6c,d and Table 1), the average lifetime (τ_avg_) monitored at the higher‐energy core emission band (435 nm) was notably shorter than that recorded at the long‐wavelength surface emission band (492 nm in TOL and 585 nm in EtOH). This temporal disparity further supports the intra‐particle energy funneling process. The highly absorbing nitrogen‐doped core effectively acts as a light‐harvesting antenna; upon excitation, excitons rapidly depopulate from the higher‐energy core state, non‐radiatively transferring (funneling) down the energy gradient into the spatially adjacent, lower‐energy oxygen surface traps prior to final photon emission [84, 85]. Consequently, the extended lifetime of the long‐wavelength emission confirms its role as the terminal radiative trap state.

**TABLE 1:** Bi‐exponential TRPL decay fitting parameters for the DAN‐CDs dispersed in non‐polar (TOL) and polar (EtOH) solvents (λ_EX_ = 375 nm), monitored at emission wavelengths of 435, 492, and 585 nm.

Sample	Solvent	Excitation (nm)	Emission (nm)	τ_1_ (ns)	A_1_ (%)	τ_2_ (ns)	A_2_ (%)	τ_avg_ (ns)
DAN‐CD	Toluene	375	435	1.085	37.7	2.455	62.3	1.94
			492	0.863	6.2	2.303	93.8	2.21
	Ethanol		435	0.494	17.7	2.220	82.3	1.92
			585	0.568	8.7	3.026	91.3	2.81

**TABLE 2:** Comparison of the TRPL decay fitting parameters for the DAN‐CDs and the DAN‐CDs‐N_2_ dispersed in different solvents (λ_EX_ = 375 nm), monitored at the core‐associated emission wavelength of 435 nm.

Sample	Solvent	Excitation (nm)	Emission (nm)	τ_1_ (ns)	A_1_ (%)	τ_2_ (ns)	A_2_ (%)	τ_avg_ (ns)
DAN‐CD^a^	Toluene	375	435	1.085	37.7	2.455	62.3	1.94
	Ethanol			0.494	17.7	2.220	82.3	1.92
DAN‐CD‐N_2_	Toluene			1.203	53.4	3.451	46.6	2.25
	Ethanol			0.571	8.2	3.547	91.8	3.30

^a^ The decay parameters for the DAN‐CDs are reproduced from Table 1 for ease of comparison.

Crucially, the distinct microenvironments and environmental sensitivities of these dual states are further corroborated by their solvent‐dependent lifetime variations (Table 1). Upon transitioning from a non‐polar aprotic solvent (TOL) to a highly polar, hydrogen‐bonding protic solvent (EtOH), the average lifetime of the 435 nm core state remains remarkably static (τ_avg_ = 1.94 ns in TOL vs. 1.92 ns in EtOH) (Figure 6c,f). This temporal consistency provides compelling kinetic evidence that the nitrogen‐doped emissive core is deeply embedded and sterically shielded within the rigid, hydrophobic sp^2^ carbon network, effectively insulating its excitons from external solvent fluctuations. In contrast, the terminal surface state exhibits a notable prolongation in its average lifetime, increasing by ∼27% from 2.21 ns (at 492 nm in TOL) to 2.81 ns (at 585 nm in EtOH) (Figure 6d,f). This extended excited‐state lifetime physically reflects the strong dipole‐dipole interactions and intermolecular hydrogen‐bonding stabilization imparted by the protic solvent onto the exposed, highly electronegative oxygen functionalities. These protic interactions rigidly coordinate the surface moieties, restricting non‐radiative vibrational relaxation and thermodynamically delaying radiative recombination [86, 87].

Furthermore, when comparing the two differently synthesized materials at the identical 435 nm core emission wavelength (Figure 6c,e and Table 2), the oxygenated DAN‐CDs exhibited consistently shorter core lifetimes than the DAN‐CDs‐N_2_ across both solvents. Because the DAN‐CDs‐N_2_ lack these highly electronegative localized surface traps, the intra‐particle energy funneling pathway is inherently precluded. This structural absence forces the core state in the inert DAN‐CDs‐N_2_ to act as the primary, uninterrupted emissive channel, thereby displaying a prolonged, intrinsic radiative lifetime [84]. Interestingly, without the dominant core‐to‐surface relaxation pathway acting as a rapid depopulation buffer, the core state in the DAN‐CDs‐N_2_ interacts more directly with the solvent matrix, leading to a much larger solvent‐dependent lifetime variation at 435 nm (from 2.25 ns in TOL to 3.30 ns in EtOH) (Figure 6e,f). Ultimately, wherever the emissive state is directly exposed to the solvent (whether it is the oxygen‐mediated surface state of the DAN‐CDs or the uninterrupted core state of the DAN‐CDs‐N_2_), dispersion in EtOH consistently prolongs the excited‐state lifetime compared to TOL. This phenomenon is attributed to the enhanced dielectric stabilization of these polar excited states via protic hydrogen‐bonding networks, which effectively restricts non‐radiative vibrational relaxation and favorably alters the kinetic balance toward radiative decay [88].

Finally, validating the exceptional efficiency of this rationally designed molecular architecture, the optimized DAN‐CDs dispersed in TOL (0.05 mg/mL) achieved an outstanding absolute PLQY of 91% when excited at the optical wavelength of 330 nm, monitoring the primary emission at 492 nm via an integrating sphere (Figure S19 and Table S3). This near‐unity PLQY is the direct physical consequence of the solvent‐free methodology. The highly rigid, nitrogen‐doped conjugated core restricts internal molecular rotation and minimizes non‐radiative vibrational energy losses [89]. Simultaneously, the uniform oxygen‐mediated surface functionalization thoroughly passivates peripheral dangling bonds that would otherwise act as non‐radiative traps [90, 91]. By successfully engineering this synergistic architecture, excitons are quantitatively funneled from the nitrogen‐doped core into a singular, highly efficient radiative surface channel, effectively suppressing non‐radiative defect pathways and maximizing overall luminous efficiency.

### 3.5 Fabrication and Performance of DAN‐CD‐Based Self‐Emissive LEDs

**FIGURE 7:**
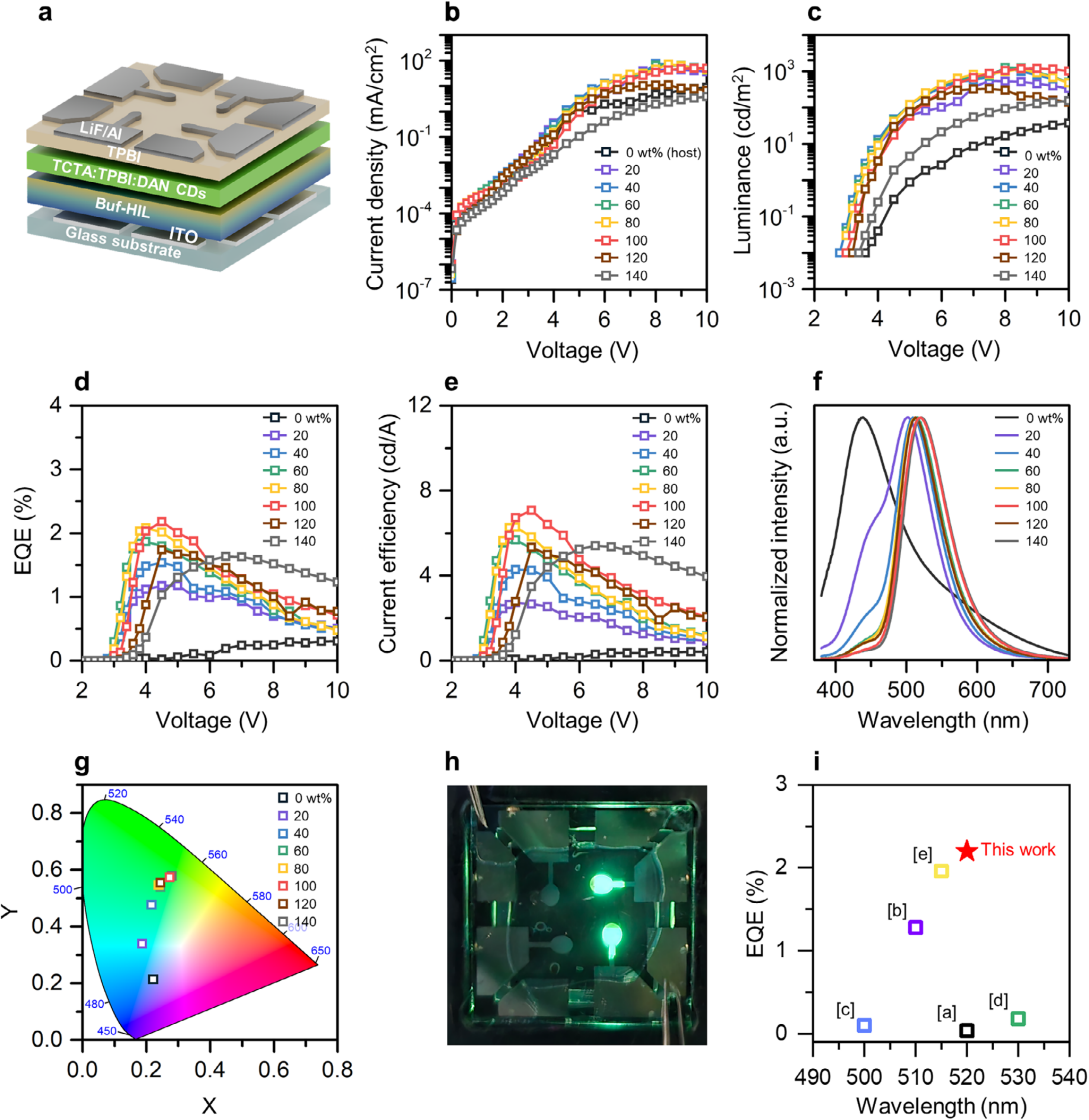
(a) Schematic illustration of the device structure. (b) Current density‐voltage, (c) luminance‐voltage, (d) EQE‐voltage, and (e) current efficiency‐voltage characteristics of the DAN‐CD‐based LEDs at varying dopant concentrations (wt.% relative to the TCTA:TPBI co‐host). (f) Normalized EL spectra and (g) CIE 1931 chromaticity coordinates of the corresponding devices. (h) Photograph of the operating optimized (100 wt.%) DAN‐CD‐based LED. (i) Comparison of the peak EQE of the optimized DAN‐CD‐based LED with previously reported green‐emissive CD‐based LEDs.

To demonstrate the practical optoelectronic viability of the green‐emissive DAN‐CDs as light emitters, we fabricated self‐emissive LEDs incorporating these CDs as luminescent dopants. The devices were constructed with a multilayer architecture consisting of glass/ITO/Buf‐HIL/TCTA:TPBI:DAN‐CD/TPBI/LiF/Al (Figure 7a). Each layer was rationally engineered to optimize charge injection and ensure a balanced flux of holes and electrons into the emissive layer. For hole injection, a buffer layer (Buf‐HIL) comprising PEDOT:PSS and PFI was utilized. Because PFI naturally self‐organizes at the surface during spin‐coating, the resulting PFI‐rich upper interface facilitates efficient hole injection into the emissive layer while simultaneously acting as an exciton‐blocking barrier to prevent non‐radiative quenching at the anode [92, 93, 94]. Within the emissive layer, the DAN‐CDs were dispersed into a bipolar TCTA:TPBI co‐host (blended at a fixed 1:1 weight ratio). This co‐host system promotes balanced internal charge transport; the TCTA component primarily assists hole mobility, whereas the TPBI component facilitates electron mobility. This synergistic architecture allows the injected charge carriers to efficiently recombine directly at the localized CD sites [95, 96, 97, 98].

To optimize device performance, the dopant concentration of the DAN‐CDs within the emissive layer was systematically varied. The concentration is expressed as a weight percentage (wt.%) relative to the total mass of the TCTA:TPBI co‐host. The LEDs were evaluated at doping levels ranging from 0 (host‐only) to 140 wt.% (Figure 7b–e and Figure S20). Across all concentrations, the devices exhibited typical diode characteristics, confirming robust charge injection and transport. The device performance demonstrated a clear parabolic trend, peaking at the 100 wt.% concentration (an equal 1:1 mass ratio of DAN‐CDs to the host matrix). This optimized LED achieved a maximum luminance of 1,202 cd/m^2^, a current efficiency of 7.0 cd/A, a power efficiency of 5.3 lm/W, and a maximum external quantum efficiency (EQE) of 2.2%. This optimal performance reflects a critical thermodynamic balance. At lower doping levels (e.g., 20–80 wt.%), the sparse density of emissive centers limits overall radiative recombination efficiency, whereas excessive loading (e.g., 120–140 wt.%) induces physical aggregation of the DAN‐CDs. This aggregation disrupts the delicate charge‐transport network of the co‐host matrix and triggers non‐radiative concentration‐induced quenching pathways.

To explicitly confirm the physical origin of the emission and evaluate the color purity of these optimized devices, the EL spectra were analyzed. As shown in Figure 7f, the undoped, host‐only control device exhibited a distinct blue emission centered at 438 nm, originating intrinsically from the TCTA:TPBI co‐host matrix. In contrast, upon doping with DAN‐CDs, this host emission was completely suppressed. All doped LEDs displayed a clear, single green emission peak centered at 520 nm, which perfectly matches the steady‐state PL spectrum of the isolated DAN‐CDs (Figure 4). This complete spectral shift verifies thorough host‐to‐guest energy transfer and confirms that the injected charge carriers successfully recombine radiatively at the targeted DAN‐CD emissive dopants. Correspondingly, the CIE 1931 chromaticity coordinates of the optimized 100 wt.% device were determined to be (0.27, 0.58), positioning the output squarely in the pure green emission region and demonstrating excellent color purity (Figure 7g,h).

The performance of the optimized 100 wt.% DAN‐CD‐based LED was benchmarked against previously reported green CD‐based LEDs (Figure 7i and Table S4). Notably, the peak EQE of 2.2% achieved herein through the solvent‐free approach stands as the highest value reported to date among LEDs based on green‐emissive CDs synthesized via thermal carbonization, a process that ensures the formation of a robust carbonaceous framework rather than low‐temperature molecular conjugation. However, achieving EQEs suitable for practical display applications requires addressing specific device‐level bottlenecks. Considering the near‐unity PLQY (91%) of the DAN‐CDs demonstrated in this work, the intrinsic radiative efficiency of the emitter is not the primary limiting factor. Instead, the EQE is determined not only by the PLQY of the emitter but also by charge balance, exciton utilization, and optical out‐coupling efficiency. We consider device‐level losses, including imperfect charge balance and possible interfacial non‐radiative recombination, to be the primary factors limiting the present EQE. Specifically, while the co‐host matrix mitigates charge trapping, the highly electronegative oxygenated surfaces of the CDs can still limit carrier hopping at high doping concentrations, leading to localized charge accumulation and non‐radiative recombination. Therefore, further optimization of the charge‐transport layers and energy‐level alignment, together with improved interfacial passivation, will provide a viable pathway toward higher EQEs while taking full advantage of the intrinsically high PLQY of the state‐of‐the‐art CDs.

Finally, to evaluate the operational stability of the DAN‐CD‐based LEDs, continuous‐operation measurements were performed at an initial luminance of 10 cd/m^2^ (corresponding to an operating current of 0.015 mA). As shown in Figure S21, the unencapsulated device exhibited a T_50_ operational lifetime (the time required for luminance to decrease to 50% of its initial value) of approximately 66 min. Although the operational lifetime currently remains limited compared with the stringent requirements for practical display applications, this result provides a quantitative assessment of the baseline device stability. As is common with early‐stage, solution‐processed nanomaterial LEDs, the primary degradation mechanism is likely not the intrinsic photobleaching of the robust CDs themselves, but rather the morphological degradation of the organic host matrix and interfacial Joule heating under continuous electrical stress. Achieving extended operational lifetimes will require specialized thin‐film encapsulation to strictly block trace moisture and oxygen, alongside optimized thermal management architectures. Consequently, this highlights advanced device packaging and host‐matrix engineering as critical targets for further device optimization.

## 4 Conclusions

In summary, we demonstrated a rational, solvent‐free strategy utilizing 2,6‐diaminonaphthalene (DAN) as a single precursor to synthesize ultrabright, green‐emissive CDs (DAN‐CDs). By eliminating the unpredictable variables associated with liquid‐phase environments, this streamlined approach enabled us to uncover a spatiotemporally resolved, dual‐pathway formation mechanism. The synthesis proceeds via the initial unhindered growth of a nitrogen‐doped conjugated core shielded within a dense autogenic precursor melt, followed by controlled, oxygen‐mediated surface passivation as the protective melt barrier progressively attenuates. This symmetric precursor effectively suppresses defect‐inducing steric hindrance, while the self‐limiting oxidative capping ensures structural uniformity and comprehensively eliminates non‐radiative defect traps. Furthermore, detailed steady‐state and time‐resolved optical characterizations explicitly uncovered the excited‐state dynamics driving their exceptional luminescence. We identified a highly efficient intra‐particle energy funneling process that quantitatively channels excitons from the higher‐energy nitrogen‐doped core down into the oxygen‐mediated surface traps. This synergistic architecture minimizes non‐radiative decay, culminating in a robust, excitation‐independent green emission characterized by a narrow FWHM (< 60 nm) and a near‐unity absolute PLQY of 91%. Finally, demonstrating their practical optoelectronic viability, we successfully deployed the optimized DAN‐CDs as emissive dopants in self‐emissive LEDs. The resulting devices delivered pure green EL with a peak EQE of 2.2% achieved herein through the solvent‐free approach, notably standing as the highest value reported to date among LEDs based on green‐emissive CDs synthesized via thermal carbonization. Ultimately, this work provides profound mechanistic insights into solvent‐free carbonization and paves the way for designing high‐performance, heavy‐metal‐free emitters for next‐generation optoelectronics and display applications.

## Author Contributions

H.P.: methodology, investigation, visualization, writing – original draft. S.H.L.: methodology, investigation, visualization, writing – original draft. S.S.: investigation, validation. S.P.: investigation, validation. S.M.J.: investigation. H.J.: validation. S.P.: methodology. Y.‐H.K.: conceptualization, methodology, supervision, funding acquisition, writing – review & editing. W.K.: conceptualization, methodology, data curation, formal analysis, supervision, funding acquisition, writing – review & editing.

## Conflicts of Interest

The authors declare no conflicts of interest.

## Supporting information




**Supporting File**: advs77885‐sup‐0001‐SuppMat.docx.

## Data Availability

The data that support the findings of this study are available from the corresponding author upon reasonable request.
