# Supplementary material for: Solvent‐Free Synthesis of Ultrabright Carbon Dots With Near‐Unity Quantum Yield for High‐Performance Light‐Emitting Diodes
Source: Adv Sci (Weinh). 2026 Sep 27:e77885. Online ahead of print. doi: 10.1002/advs.77885 (PMC13616399; doi:10.1002/advs.77885)
Supplement: Supplementary file 1 [file ADVS-9999-e77885-s001.docx]

**Supplementary Information**

**Solvent‐Free Synthesis of Ultrabright Carbon Dots with Near‐Unity Quantum Yield for High‐Performance Light‐Emitting Diodes**

Hyeonjin Park^a^, Su Hwan Lee^b^, Sejeong Seo^a^, Soye Park^a^, Soo min Ji^c^, Hyunda Jo^a^, Sehyeon Park^a^, Young**‐**Hoon Kim^b,c,*^, Woosung Kwon^a,d,e*^

^a^Department of Chemical and Biological Engineering, Sookmyung Women’s University, 100 Cheongpa**‐**ro 47**‐**gil, Yongsan**‐**gu, Seoul, 04310, Republic of Korea

^b^Department of Energy Engineering, Hanyang University, 222 Wangsimni**‐**ro, Seongdong**‐**gu, Seoul 04763, Republic of Korea

^c^Department of Battery Engineering, Hanyang University, 222 Wangsimni**‐**ro, Seongdong**‐**gu, Seoul 04763, Republic of Korea

^d^Institute of Advanced Materials and Systems, Sookmyung Women’s University, 100 Cheongpa**‐**ro 47**‐**gil, Yongsan**‐**gu, Seoul, 04310, Republic of Korea

^e^Trion Co. Ltd., 40 Imi-ro, Uiwang-si, Gyeonggi-do, 16006, Republic of Korea

^*^Corresponding authors.

Woosung Kwon

Tel.: +82‐2‐2077‐7398; Fax: +82‐2‐2077‐7459; Email: [wkwon@sookmyung.ac.kr](mailto:wkwon@sookmyung.ac.kr)

Young**‐**Hoon Kim

Tel.: +82‐2‐2220‐2393; Fax: +82‐2‐2220‐3119; Email: [younghoonkim@hanyang.ac.kr](mailto:younghoonkim@hanyang.ac.kr)

**Table S1.** Comparison of performance metrics among solvent-free aromatic diamine-based CDs.

| Precursor | EM peak (nm) | FWHM (nm) | PLQY (%) | EQE (%) | Ref |
| --- | --- | --- | --- | --- | --- |
| *p*-phenylenediamine | 615 | 29 | 12.8 | NA | [24] |
| *o*-phenylenediamine | 585 | 34 | NA | NA | [24] |
| *m*-phenylenediamine | 575 | 32 | NA | NA | [24] |
| *o*-phenylenediamine, KCL | 442-621 | NA | 23-56 | NA | [37] |
| *o*-phenylenediamine, ZnSO_4_ | 548 | NA | 53 | NA | [38] |
| *p*-phenylenediamine, ZnSO_4_ | 606 | NA | 7 | NA | [38] |
| *m*-phenylenediamine, Urea | 420 | 90 | 11 | NA | [39] |
| *p*-phenylenediamine,  Urea | 630 | 130 | 6 | NA | [39] |
| 2,6-diaminonaphthalene | 492 | 60 | 91 | 2.2 | This Work |

**Fig.** **S1.** Proposed detailed reaction mechanisms. (a) Amine-mediated intermolecular coupling process for the formation of the carbonaceous core. (b) Oxygen-mediated functionalization process at the peripheral edge sites. (c) Kinetic simulation of solid-state DAN carbonization. The left axis tracks the depletion of the DAN precursor (or the decrease in the diffusion barrier thickness, dashed red line) and the corresponding increase in surface oxidation (dashed green line). The right axis (solid blue line) illustrates the physical growth of the DAN-CD radius over reaction time. Governed by a precursor-shielded growth mechanism, the rapid surface oxidation initiates a self-limiting effect. The shielding coefficient *β* was calculated using the experimentally observed mean particle radius of ~4 nm (from TEM and DLS analysis) as a terminal boundary condition. The model is highly sensitive to this parameter. A value of *β* that is too low simulates premature barrier collapse and rapid oxidation, yielding critically undersized particles. Conversely, an excessively high *β* simulates delayed passivation and runaway core growth. Constraining *β* utilizing the final physical radius ensures that the simulated temporal onset of the oxidation spike is fundamentally tied to experimental structural reality.

**Fig.** **S2.** (a) FTIR and (b) ^1^H NMR spectra comparing the pristine DAN precursor, the air‐synthesized DAN‐CDs, and the inert‐synthesized DAN‐CDs‐N_2_ (carbonized at 300 °C).

**Fig.** **S3.** FTIR spectra tracking the sequential structural evolution of the pristine DAN precursor and reaction intermediates obtained at distinct spatiotemporal stages (200 °C, 250 °C, and 300 °C for 0, 1, 3, 6, and 24 h). The delayed emergence of C−O and O−H bands validates the initial suppression of oxidation, while their rapid intensification after 3 hours at 300 °C confirms the onset of the massive oxidative capping phase.

**Fig.** **S4.** XPS survey spectra monitoring the elemental composition evolution throughout the synthesis process: (a) DAN precursor, (b) 200 °C, (c) 250 °C, (d) 300 °C: 0 h, (e) 300 °C:1 h, (f) 300 °C:3 h, (g) 300 °C:6 h, and (h) 300 °C:24 h.

**Table S2.** Atomic compositions (C, N, and O atomic percentages) of the pristine DAN precursor and reaction intermediates at different heating temperatures and time intervals, determined via quantitative XPS survey analysis. The data highlights the minimal oxygen exposure during the initial stages (200-250 °C), the sudden onset of oxidative capping (peaking at 15.95% O at 300 °C:6 h), and final structural stabilization following partial thermal decarboxylation (300 °C, 24 h).

| Sample | Atomic Compositions (%) | | |
| --- | --- | --- | --- |
|  | C | N | O |
| Precursor | 81.76 | 16.45 | 1.79 |
| 200 °C | 81.50 | 15.90 | 2.60 |
| 250 °C | 81.88 | 14.18 | 3.97 |
| 300 °C:0 h | 80.80 | 13.85 | 5.35 |
| 300 °C:1 h | 80.93 | 14.93 | 4.14 |
| 300 °C:3 h | 78.84 | 14.20 | 6.96 |
| 300 °C:6 h | 72.67 | 11.38 | 15.95 |
| 300 °C:24 h | 77.32 | 13.63 | 9.05 |

**Fig.** **S5.** High-resolution C 1s XPS spectra of the samples at progressive reaction stages: (a) pristine DAN precursor, (b) 200 °C, (c) 250 °C, (d) 300 °C:0 h, (e) 300 °C:1 h, (f) 300 °C:3 h, (g) 300 °C:6 h, and (h) 300 °C:24 h.

**Fig.** **S6.** High-resolution N 1s XPS spectra of the samples at progressive reaction stages: (a) pristine DAN precursor, (b) 200 °C, (c) 250 °C, (d) 300 °C:0 h, (e) 300 °C:1 h, (f) 300 °C:3 h, (g) 300 °C:6 h, and (h) 300 °C:24 h.

**Fig.** **S7.** High-resolution O 1s XPS spectra of the samples at progressive reaction stages: (a) pristine DAN precursor, (b) 200 °C, (c) 250 °C, (d) 300 °C:0 h, (e) 300 °C:1 h, (f) 300 °C:3 h, (g) 300 °C:6 h, and (h) 300 °C:24 h.

**Fig.** **S8.** DLS size distributions of the pristine DAN precursor and intermediate samples collected at progressive reaction stages: (a) pristine DAN precursor, (b) 200 °C, (c) 250 °C, (d) 300 °C:0 h, (e) 300 °C:1 h, (f) 300 °C:3 h, (g) 300 °C:6 h, and (h) 300 °C:24 h.

**Fig.** **S9.** Wide-field TEM images of the intermediate DAN-CDs collected at progressive reaction stages: (a) 200 °C, (b) 250 °C, (c) 300 °C:0 h, (d) 300 °C:1 h, (e) 300 °C:3 h, and (f) 300 °C:24 h. All images were acquired at the same magnification to illustrate the macroscopic structural transition from bulk amorphous aggregates to segregated nanoparticles.

**Fig.** **S10.** TEM images of the intermediate DAN-CDs collected at progressive reaction stages: (a) 200 °C, (b) 250 °C, (c) 300 °C:0 h, (d) 300 °C:1 h, (e) 300 °C:3 h, and (f) 300 °C:24 h.

**Fig.** **S11.** HR-TEM images of the intermediate DAN-CDs collected at progressive reaction stages: (a) 300 °C:0 h, (b) 300 °C:1 h, (c) 300 °C:3 h, and (d) 300 °C:24 h. The images depict the continuous nanoscale size reduction and structural refinement of the CDs.

**Fig.** **S12.** HR-TEM images of the intermediate DAN-CDs collected at progressive reaction stages: (a) 300 °C:0 h, (b) 300 °C:1 h, (c) 300 °C:3 h, and (d) 300 °C:24 h. The appearance of well-defined crystalline lattice fringes visually confirms the maturation of the extensively conjugated carbonaceous core during the final stabilization phase.

**Fig.** **S13.** (a) PL emission contour map and (b) corresponding excitation‐independent PL emission spectra of the pristine DAN precursor.

**Fig.** **S14.** Normalized UV‐visible absorption spectra of the DAN precursor and the DAN‐CDs‐N_2_, alongside the PL excitation spectrum of the DAN‐CDs‐N_2_ (λ_EM_ = 435 nm).

**Fig. S15.** Reversibility of the solvent-induced aggregation of the DAN-CDs. (a, c) Normalized PL spectra and (b, d) DLS size distributions of the pristine DAN-CDs in TOL, aggregated in a protic solvent, and redispersed back into TOL. The protic solvents utilized for the solvent-exchange experiments are (a, b) EtOH and (c, d) DW.

**Fig.** **S16.** UV‐visible absorption spectra of the pristine DAN precursor and the DAN‐CDs synthesized at different carbonization temperatures (100, 200, 300, and 400 °C).

**Fig. S17.** UV‐visible absorption spectra of the CDs synthesized using different DAN isomers: 1,5‐DAN, 1,8‐DAN, and 2,3‐DAN. The data for the precursor (2,6‐DAN) and the resulting (2,6‐)DAN‐CDs are provided as a comparative baseline.

**Fig. S18.** (a, c, e) PL emission contour maps and (b, d, f) corresponding PL emission spectra of the CDs synthesized using different DAN isomers: (a, b) 1,5‐DAN, (c, d) 1,8‐DAN, and (e, f) 2,3‐DAN.

**Fig.** **S19.** Absolute PL spectrum of the optimized DAN‐CDs dispersed in TOL (0.05 mg/mL), measured utilizing an integrating sphere (λ_EX_ = 330 nm).

**Table S3.** Summary table of the instrumental parameters used for the absolute PLQY calculation.

| Sample | PLQY | Absorption | EX peak | EX peak count | EM peak | EM peak count |
| --- | --- | --- | --- | --- | --- | --- |
| DAN‐CD | 91% | 0.06 | 330 nm | 1.93×10^6^ | 492 nm | 6.27×10^3^ |

**Fig. S20.** Power efficiency-voltage characteristics of the DAN-CD-based LEDs at varying dopant concentrations (wt% relative to the TCTA:TPBI co-host).

**Table S4.** Performance comparison of the DAN-CD-based LED developed in this work against previously reported representative green-emissive LEDs based on CDs synthesized via thermal carbonization.

| **Materials** | **λ_EL_ (nm)** | **EQE (%)** | **Ref.** | |
| --- | --- | --- | --- | --- |
| TPA-CD/CBP | 520 | 0.035 | [a] | Chem. Eng. J. 470, 144112 (2023) |
| GQD/TCTA,TPBI | 510 | 1.28 | [b] | Sci. Rep. 6, 24205 (2016) |
| GAD/CBP | 500 | 0.10 | [c] | Nano Lett.  14, 1306–1311 (2014) |
| CDs/PVK | 530 | 0.18 | [d] | J. Phys. Chem. Lett. 12, 12107–12113 (2021) |
| CDs/PVK | 515 | 1.96 | [e] | Nano Lett. 23, 8794–8800 (2023) |
| CQD/PVK | 510 | NA | [f] | Nat. Commun. 9, 2249 (2018) |
| DAN-CD/ TCTA:TPBI | 520 | 2.20 | This work | ‐ |

**Fig.** **S21.** Operational stability of the optimized 100 wt% DAN-CD-based LED measured under continuous operation at an initial luminance of 10 cd/m^2^ and an initial operating current of 0.015 mA.

**References**

[a] K. Xu, M. Zheng, H. Ma, B. Zhao, H. Jia, C. Zhang, Z. Ren, C. Li, Z. Tan, Solvent-free synthesis of oil-soluble fluorescent carbon dots for bright photoluminescent and electroluminescent light-emitting diodes, Chem. Eng. J. 470 (2023) 144112, https://doi.org/10.1016/j.cej.2023.144112

[b] W. Kwon, Y.-H. Kim, J.-H. Kim, T. Lee, S. Do, Y. Park, M.S. Jeong, T.-W. Lee, S.-W. Rhee, High color-purity green, orange, and red light-emitting diodes based on chemically functionalized graphene quantum dots, Sci. Rep. 6 (2016) 24205, https://doi.org/10.1038/srep24205

[c] W. Kwon, Y.-H. Kim, C.-L. Lee, M. Lee, H.C. Choi, T.-W. Lee, S.-W. Rhee, Electroluminescence from graphene quantum dots prepared by amidative cutting of tattered graphite, Nano Lett. 14 (2014) 1306–1311, https://doi.org/10.1021/nl404281h

[d] Z. Zheng, Z. Liu, Y. Ding, M. Chen, P. Lv, A. Tang, F. Wang, L. Guan, X. Li, B. Liang, Structural engineering toward high monochromaticity of carbon dots-based light-emitting diodes, J. Phys. Chem. Lett. 12 (2021) 12107–12113, https://doi.org/10.1021/acs.jpclett.1c03786

[e] B. Wang, H. Wang, Y. Hu, G.I.N. Waterhouse, S. Lu, Carbon dot based multicolor electroluminescent LEDs with nearly 100% exciton utilization efficiency, Nano Lett. 23 (2023) 8794–8800, https://doi.org/10.1021/acs.nanolett.3c02271

[f] F. Yuan, T. Yuan, L. Sui, Z. Wang, Z. Xi, Y. Li, X. Li, L. Fan, Z. Tan, A. Chen, M. Jin, S. Yang, Engineering triangular carbon quantum dots with unprecedented narrow bandwidth emission for multicolored LEDs, Nat. Commun. 9 (2018) 2249, https://doi.org/10.1038/s41467-018-04635-5
